# Multiprotein *E. coli* SSB–ssDNA complex shows both stable binding and rapid dissociation due to interprotein interactions

**DOI:** 10.1093/nar/gkaa1267

**Published:** 2021-01-12

**Authors:** M Nabuan Naufer, Michael Morse, Guðfríður Björg Möller, James McIsaac, Ioulia Rouzina, Penny J Beuning, Mark C Williams

**Affiliations:** Department of Physics, Northeastern University, Boston, MA 02115, USA; Department of Physics, Northeastern University, Boston, MA 02115, USA; Department of Physics, Northeastern University, Boston, MA 02115, USA; Department of Chemistry and Chemical Biology, Northeastern University, Boston, MA 02115, USA; Department of Chemistry and Biochemistry, Ohio State University, Columbus, OH 43210, USA; Department of Chemistry and Chemical Biology, Northeastern University, Boston, MA 02115, USA; Department of Physics, Northeastern University, Boston, MA 02115, USA

## Abstract

*Escherichia coli* SSB (*Ec*SSB) is a model single-stranded DNA (ssDNA) binding protein critical in genome maintenance. *Ec*SSB forms homotetramers that wrap ssDNA in multiple conformations to facilitate DNA replication and repair. Here we measure the binding and wrapping of many *Ec*SSB proteins to a single long ssDNA substrate held at fixed tensions. We show *Ec*SSB binds in a biphasic manner, where initial wrapping events are followed by unwrapping events as ssDNA-bound protein density passes critical saturation and high free protein concentration increases the fraction of *Ec*SSBs in less-wrapped conformations. By destabilizing *Ec*SSB wrapping through increased substrate tension, decreased substrate length, and protein mutation, we also directly observe an unstable bound but unwrapped state in which ∼8 nucleotides of ssDNA are bound by a single domain, which could act as a transition state through which rapid reorganization of the *Ec*SSB–ssDNA complex occurs. When ssDNA is over-saturated, stimulated dissociation rapidly removes excess *Ec*SSB, leaving an array of stably-wrapped complexes. These results provide a mechanism through which otherwise stably bound and wrapped *Ec*SSB tetramers are rapidly removed from ssDNA to allow for DNA maintenance and replication functions, while still fully protecting ssDNA over a wide range of protein concentrations.

## INTRODUCTION

Single-stranded DNA binding proteins (SSBs) rapidly sequester and protect transiently formed single-stranded DNA (ssDNA) segments during genome maintenance (1–12). They exhibit high affinity ssDNA binding and may also play regulatory roles by interacting with other proteins involved in genome maintenance (13,14). The SSB from *Escherichia coli* (*Ec*SSB) is a model SSB that has been extensively studied.

An *Ec*SSB monomer (molecular weight 19 kDa), consists of an N-terminal domain containing an oligonucleotide binding (OB) fold, a C-terminal domain (CTD) with a conserved 9-amino acid acidic tip, and a poorly conserved intrinsically disordered linker (IDL) (15–19). The N-terminal OB domain mediates both inter-protein interactions to form tetramers (which are referred to as *Ec*SSB henceforth), as well as high-affinity DNA binding. *Ec*SSB was shown to exhibit high cooperativity in certain ssDNA binding conformations, which is eliminated by truncating or replacing the IDL or acidic tip, as well as by mutating the ‘bridge interface’ that links adjacent SSB tetramers through an evolutionarily conserved surface near the ssDNA-binding site (2,6,8,12,18,20–22). *Ec*SSB can bind ssDNA with multiple conformations that wrap the ssDNA substrates to different degrees (8,23–26). The distinct binding modes of *Ec*SSB are identified based on the number of nucleotides (n) occluded by the tetramer upon binding to ssDNA. Solution conditions such as the salt composition and concentration, protein density, as well as template tension have been shown to affect the stability of these distinct binding modes (8,23–27). Importantly, high cooperativity of binding appears to be typical of the low-salt *Ec*SSB–ssDNA complexes (<20 mM NaCl, <1 mM MgCl_2_), when only two out of the four OB-fold domains of the *Ec*SSB tetramer are associated with ssDNA (28). Moreover, it appears that *Ec*SSB mutants that lack cooperative behavior are fully functional for replication in cells and are able to complement deletion of the *ssb* gene in *E. coli* (22). Thus far, three stable or semi-stable ssDNA binding modes (*Ec*SSB_35_, *Ec*SSB_56_, and *Ec*SSB_65_, where the subscript indicates the number of nucleotides occupied by the protein) have been identified and well characterized. Additionally, a recent study by Suksombat *et al.* (27), observing single *Ec*SSB tetramers binding a 70 nt long poly dT ssDNA substrate held by optical tweezers, measured a less wrapped state consistent with ∼17 nt bound by *Ec*SSB (noted hereafter *Ec*SSB_17_). This study also found that higher applied tensions favored less wrapped states (fewer nt bound per protein), with only the *Ec*SSB_17_ state observed at tensions above 8 pN. X-ray crystallographic structural studies revealed a model for the *Ec*SSB_65_ binding topology in which the ssDNA is fully wrapped through the association of all four *Ec*SSB subunits (16). While the precise topologies of the other binding modes have not been structurally resolved, the *Ec*SSB_17_ and *Ec*SSB_35_ states are geometrically consistent with the wrapped ssDNA directly binding to two and three of the domains of the *Ec*SSB tetramer, respectively. However, there have been limited reports of *Ec*SSB binding segments of ssDNA that should be too short to accommodate any of these wrapped states. First, a sedimentation experiment observed *Ec*SSB binding 8 nt poly dT oligos with a stoichiometry of more than three oligos per tetramer, suggesting each individual domain must be capable of binding short ssDNA fragments (29). Second, a single molecule FRET experiment observed that the addition of a poly(dT) ssDNA overhang to a hairpin substrate significantly enhanced the ability of *Ec*SSB to disrupt and bind the otherwise stable hairpin, suggesting *Ec*SSB can transiently bind the short ssDNA overhang before wrapping the ssDNA contained in the hairpin (30). However, both these experiments measured an effective binding affinity between *Ec*SSB and these short (∼8 nt) ssDNAs to be ∼10 μM, compared to the <1 nM affinity of the wrapped states, which likely explains the difficulty in other experiments of observing this mode due to its extremely low stability on an unsaturated ssDNA substrate.

Recent single molecule FRET experiments have revealed the dynamic equilibrium between well-defined *Ec*SSB functional and structural states (31), and the ability of the tetramer to diffuse quickly along the ssDNA substrate while maintaining its wrapped conformation (32). Additionally, fluorescent imaging of *Ec*SSB–ssDNA complexes have been able to resolve kinetics of *Ec*SSB binding and wrapping, including a fast, concentration-dependent rate of initial binding (33), an even faster concentration-independent rate of wrapping (34), much slower binding of additional protein to an ssDNA substrate with *Ec*SSB already bound (35), and the direct transfer of an *Ec*SSB tetramer between two different ssDNA substrates (36). Nevertheless, several longstanding questions on *Ec*SSB function remain ambiguous, especially with respect to its collective binding dynamics and kinetics. To this end, we directly observe the binding and wrapping dynamics of many *Ec*SSB proteins on a long ssDNA substrate, especially after abrupt introduction or removal of free protein, resulting in *Ec*SSB reorganization. We utilize an optical tweezers system, which allows for the direct real-time measurement of collective *Ec*SSB binding and wrapping dynamics through ssDNA extension and the application of force to bias these wrapping states and isolate the kinetics of transitions that are otherwise difficult to observe. This includes the first extensive characterization of an *Ec*SSB state that does not wrap ssDNA by binding to the substrate by only a single OB-fold domain. This complex likely serves as a transition state through which free *Ec*SSB initially binds ssDNA before wrapping and before wrapped *Ec*SSB is able to release and completely dissociate from ssDNA. We also identify a critical point of protein saturation, above which *Ec*SSB tetramers bind in a competitive fashion, destabilizing the wrapping and binding of their neighbors. These interactions are critical to the seemingly paradoxical function of *Ec*SSB. On one hand, it must have high affinity and stable binding while occupying up to 65 nt of ssDNA per tetramer to allow *Ec*SSB to fully protect long stretches of ssDNA even under conditions of low free protein concentration. On the other hand, during DNA processing events, *Ec*SSB must be rapidly removed as the ssDNA segment shrinks in length. Based on the results from this study, we propose a mechanism for rapid self-regulation of *Ec*SSB density to continuously provide optimal ssDNA coverage during genomic maintenance.

## MATERIALS AND METHODS

### Preparation of DNA substrates and proteins

For the optical tweezer experiments, an 8.1 kbp dsDNA construct with digoxigenin (DIG) and biotin labeled ends with a free 3′ end was constructed as previously described (37). Vector pBACgus11 (gift from Borja Ibarra) was linearized through double digestion using restriction enzymes SacI and BamHI (New England Biolabs, NEB). A dsDNA handle with digoxigenin (DIG) labeled bases with a complementary end to the BamHI sequence was PCR amplified (38). The DIG handle and a biotinylated oligo (Integrated DNA Technologies, IDT) were annealed to the overhangs produced by BamHI and SacI and ligated using T4 DNA ligase (NEB).

For the AFM experiments, a hybrid dsDNA–ssDNA construct was produced, which enables accurate detection of protein binding to an ssDNA substrate (39). A PCR amplified dsDNA segment from pUC19 was digested by BamHI and ligated to an oligo with a complementary end (IDT) using T4 DNA ligase. The final product consisted of 100 bp of dsDNA with an 8 nt long poly dT tail.

WT *Ec*SSB and T7 DNA polymerase were purchased (NEB). The plasmid encoding WT *Ec*SSB pEAW134 was a gift from Dr Mark Sutton of the University at Buffalo. The *Ec*SSB_H55Y_ variant was constructed using Quikchange site-directed mutagenesis (Agilent) and mutagenic oligonucleotides. Recombinant protein *Ec*SSB_H55Y_ was expressed in *E. coli* BL21 Tuner cells in 1 l Luria Broth with ampicillin (100 μg/ml). After the cells reached an OD_600_ of ∼0.7 expression was induced by adding IPTG to a final concentration of 1 mM and shaking at 220 rpm for 4 h at 30°C. Purification was carried out based on the protocols outlined by Lohman *et al.* with some modification (40). All subsequent steps were carried out at 4°C or on ice. For WT *Ec*SSB cells were collected by centrifugation and resuspended in 20 ml of buffer containing 50 mM Tris pH 8.3, 200 mM NaCl, 15 mM Spermidine, 1 mM EDTA, 100 μM PMSF and 10% sucrose. Lysis was carried out via sonication and the addition of lysozyme. Cells containing *Ec*SSB_H55Y_ were handled similarly except for an increase in salt to 400 mM NaCl to induce the alternate DNA binding mode *Ec*SSB_65_ to compensate for the reduced binding affinity of the H55Y variant (41). The collected supernatant was subjected to Polymin P (Sigma Aldrich) precipitation by adding a 5% solution dropwise to a final concentration of 0.4%. Stirring was continued for 20 min before centrifugation at 10 000 × g for 20 min. The resulting pellet was collected and resuspended gently in 50 mM Tris pH 8.3, 400 mM NaCl, 1 mM EDTA and 20% glycerol to the initial fraction volume over 60 min followed by centrifugation at 10 000 × g for 20 min. *Ec*SSB was precipitated from the collected supernatant by slowly adding ammonium sulfate (Sigma Aldrich) with stirring to a final concentration of 150 g/l and manually stirring for an additional 30 min followed by centrifugation at 24,000 × g for 30 min. The resulting pellet was resuspended in 50 mM Tris pH 8.3, 300 mM NaCl, 1 mM EDTA and 20% glycerol at 0.9× fraction volume. Purity was examined by SDS-PAGE and concentration determined by Bradford assay before loading onto a 20 ml spin column packed with 5 ml ssDNA–cellulose (Sigma Aldrich D8273). The column with SSB containing fractions was sealed and incubated for 60 min with gentle rocking. Washing and elution were carried out by centrifugation at 1000 × g and the duration of each centrifugation event was determined prior to loading the protein in order to prevent drying the column. The buffer used for wash and elution steps was 50 mM Tris pH 8.3, 1 mM EDTA, 20% glycerol and NaCl at 300 mM, 600 mM or 2 M. After allowing the column to drain it was washed with 10 CV of 300 mM NaCl buffer, then 10 CV of 600 mM NaCl buffer followed by elution with 10 CV of 2 M NaCl. Fractions were evaluated by SDS-PAGE and the 2 M NaCl elution fractions containing SSB were pooled before concentrating by ammonium sulfate precipitation at 225 g/l. The resulting pellet is then resuspended in 50 mM Tris pH 8.3, 300 mM NaCl, 1 mM EDTA, 1 mM β-Mercaptoethanol and 50% glycerol to the desired concentration, as determined by Bradford assay.

### Optical tweezers

The 8.1 kbp dsDNA construct with a primer-template junction at one terminus was tethered between 2 μm anti-digoxigenin and 3 μm streptavidin functionalized beads (Spherotech) held in place by a micropipette tip and a dual beam optical trap, respectively. The micropipette tip was moved by a piezo electric stage with 0.1 nm precision to change the extended length of the DNA while the deflection of the laser trap was measured to calculate the force exerted on the trapped bead and thus the tension along the DNA. Additionally, a bright-field image of the two beads was recorded at 40× magnification. The DNA was held in a single fluidic chamber fed upstream by multiple inlet channels driven by air pressure and controlled by clamp valves. The instrument was controlled via a NI-DAQ interface and custom software compiled with LabWindows (National Instruments). In order to create an ssDNA binding template, T7 DNA polymerase (T7DNAp) was introduced into the sample and the DNA was held at a constant force of 50 pN to trigger exonucleolysis (42) and completely digest one strand to produce a long ssDNA. After thorough rinsing of the T7DNAp reaction buffer, DNA was held at fixed forces in a buffer containing 10 mM HEPES, 50 mM Na^+^, pH 7.5, except where specifically noted. Then *Ec*SSB was introduced to the cell and the position of the bead was continuously adjusted via a force feedback loop to maintain constant DNA tension. Free *Ec*SSB in the solution was removed by replacing the protein solution with a protein free buffer. After data acquisition, the relative distance between the beads was calculated using the bright-field images and compared to the extension of the DNA as calculated by the position of the piezo electric state. This comparison allows to correct for long term thermal drifts of the flow cell system. All the data were analyzed using custom code written in MATLAB (MathWorks). All experimental conditions were performed with *N* ≥ 3 replicates, using a new ssDNA substrate and dilution of *Ec*SSB for each replicate. Experimental data were analyzed, and differential equations based on the presented model were numerically solved using custom written MATLAB (MathWorks) scripts.

### AFM imaging

*Ec*SSB and dsDNA–ssDNA hybrid constructs were incubated at an equimolar ratio (5 nM) in a buffer containing 10 mM Na^+^, 10 mM Mg^2+^ and 10 mM HEPES, pH 7.5. The sample was deposited on an APTES coated functionalized mica surface (43) and then imaged in fluid using peak force tapping mode (Bruker). Images were analyzed using Gwyddion software and height thresholds of 0.5 and 1.5 nm were used to identify dsDNA markers and *Ec*SSB tetramers, respectively.

## RESULTS

### Competitive ssDNA binding assay for *Ec*SSB

**Figure 1.**
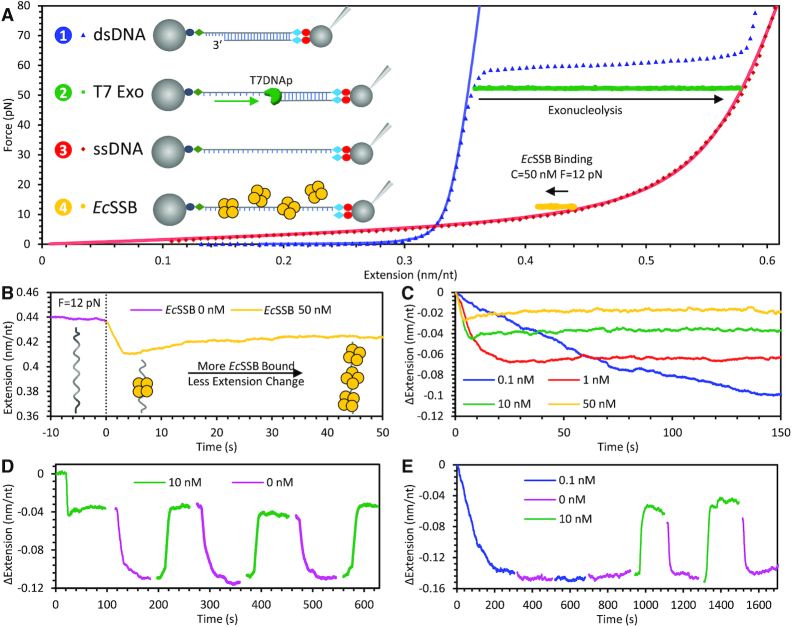
Experimental procedure to measure *Ec*SSB–ssDNA binding dynamics. (**A**) An 8.1 kbp dsDNA with a recessed 3′ end is tethered between two functionalized beads (step 1, blue). One bead is held by a glass micropipette tip which is moved by a piezo electric stage to extend the DNA. The other bead is held in a stationary dual beam optical trap, the deflection of which measures the force acting on the ssDNA substrate. The dsDNA is incubated with T7 DNA polymerase and held at 50 pN to trigger exonucleolysis to digest the bottom strand (step 2, green), resulting in a long ssDNA molecule (step 3, red). The ssDNA is then held at a constant force and incubated with varying concentrations of *Ec*SSB (step 4, yellow). Force-extension curves for dsDNA and ssDNA are fit to the WLC and FJC polymer models, respectively. T7 polymerase strand digestion is registered as an increase in the DNA extended length while held at constant force. *Ec*SSB binding results in a decrease in DNA extension. (**B**) The extension of ssDNA during *Ec*SSB incubation is plotted as a function of time. The ssDNA extension at equilibrium is shorter than bare ssDNA, but longer than the minimum extension achieved immediately after the introduction of *Ec*SSB. (**C**) Reduction in the *Ec*SSB concentration in the solution increases the net extension change of the *Ec*SSB–ssDNA complex. (**D**) *Ec*SSB concentration jump experiments showing that removal of the free *Ec*SSB in the solution after initial incubation results in an extension decrease that stably equilibrates (∼100 s) on a maximally wrapped conformation. Re-introducing *Ec*SSB solution to this equilibrated complex results in an increase in the complex extension, and oscillations between these two extension values are repeatable through changes in free protein concentration. (**E**) When ssDNA is incubated with low *Ec*SSB concentration, there is no additional change in extension associated with the removal of free protein. Increasing *Ec*SSB concentration, however, still increases the ssDNA extension change.

To characterize the collective ssDNA binding and wrapping kinetics of *Ec*SSB, we generated an 8.1 knt long ssDNA substrate in an optical tweezers system (Figure 1A). The ssDNA was then stretched and maintained at a tension of 12 pN via a force feedback loop. We initially performed experiments at a tension of 12 pN for direct comparison, as previous single molecule experiments observed that higher ordered wrapped states (>*Ec*SSB_17_) were inhibited at such force (27). Initially, a protein-free buffer (50 mM Na^+^, 10 mM HEPES, pH 7.5, unless otherwise stated) was constantly flowed into the fluidic channel (∼1 μl/s with a linear flow speed ∼200 μm/s). The flow was then switched to a fixed *Ec*SSB concentration in the same buffer (Figure 1B), with complete exchange of the solution conditions surrounding the DNA occurring on the timescale of ∼1 s. While the tension along the ssDNA was maintained, the binding of *Ec*SSB to the ssDNA resulted in a change in ssDNA extension. We observed a biphasic binding profile at saturating *Ec*SSB concentrations (≥1 nM) wherein a rapid shortening of the ssDNA was followed by a slower elongation that equilibrates to an extension less than that of a protein-free ssDNA molecule. Both the initial rapid ssDNA shortening and its subsequent partial recovery of extension occur over a longer timescale as the protein concentration is decreased (Figure 1C). At sufficiently low concentration (∼0.1 nM), the second phase disappears completely, and the ssDNA compacts at a single exponential rate. Additionally, the amplitude of the final, equilibrium change in ssDNA extension induced by *Ec*SSB decreases as free protein concentration in solution is increased.

We next measured how *Ec*SSB already bound to the ssDNA substrate reacts to changes in the surrounding free protein concentration. For each initial *Ec*SSB concentration, after the ssDNA–*Ec*SSB complex reached an equilibrated length, free protein was rapidly removed from the flow cell by flowing in protein-free buffer (Figure 1D). This resulted in a sudden decrease in ssDNA extension, which was then stable over the timescale of our observation (up to 100s of seconds). When we then reintroduced free protein into the sample, the ssDNA extension increased, returning to the same equilibrium value achieved during the first incubation. Further, this entire process of ssDNA extension change through the addition and removal of *Ec*SSB from the sample is repeatable over many cycles, with the ssDNA extension reaching the same equilibrium as previous cycles. In contrast, when the ssDNA is incubated with sufficiently low *Ec*SSB concentration (0.1 nM), the ssDNA reaches and maintains its maximum compaction during incubation, no biphasic extension increase is observed, and removal of free protein did not result in further compaction of the substrate (Figure 1E). A subsequent increase of free protein concentration, however, did trigger ssDNA extension (consistent with initial incubation at high concentration). Thus, the observed increases and decreases in ssDNA extension when the free *Ec*SSB concentration is changed are fully reversible and the ssDNA–*Ec*SSB complex will equilibrate to a set length based on the current free protein conditions, without regard to previous conditions.

Inferring the wrapping kinetics of many *Ec*SSBs on a single ssDNA substrate is greatly complicated by the multiple modes of *Ec*SSB wrapping. However, Suksombat *et al.* (27), showed that for a single protein on a ssDNA substrate at sufficient tension (>8 pN), the *Ec*SSB_35_, *Ec*SSB_56_, *Ec*SSB_65_ states are no longer observed. Instead, minimal ssDNA compaction was observed (∼2 nm at the forces we are measuring), consistent with an effective binding site size of ∼17 nucleotides (*Ec*SSB_17_). Thus, in terms of overall ssDNA compaction along the entire substrate, our low concentration results are consistent with this previous single protein experiment (2 nm/17 nt ≈ 0.1 nm/nt). This suggests that at low protein concentrations, the many proteins saturating the 8.1 knt ssDNA template are each binding the substrate in the same conformation that a single protein would bind in isolation. We further test this agreement by examining our data for single molecule wrapping events when incubating 8.1 knt ssDNA with the lowest *Ec*SSB concentration (50 pM) where we reliably observe near-saturated binding (Supplementary Figure S1). While this system is less optimized for single molecule measurements in comparison to the 70 nt poly dT ssDNA template used in Suksombat *et al.* (our 8.1 knt 50% GC ssDNA is highly dynamic and multiple wrapping events can occur simultaneously even at low concentration), we are still able to resolve a peak at ∼2 nm in the distribution of ssDNA compaction events. Moreover, at a lower ssDNA tension of 7 pN, we measure larger compaction events with a peak around ∼5 nm (Supplementary Figure S2), which is also consistent with experiments at similar force in Suksombat *et al.* (27).

In contrast, at high concentrations, we observed that bound *Ec*SSB is unable to compact ssDNA to the same degree, suggesting interprotein interactions are somehow interfering with even this minimal *Ec*SSB_17_ wrap state. As we will provide evidence for in the following sections, this is likely due to *Ec*SSB’s ability to bind ssDNA in a completely unwrapped state that sterically inhibits other proteins from wrapping. Thus, at 12 pN tension, we can characterize our competitive binding assays based on how many *Ec*SSB tetramers are in the 17 nt wrap state (*Ec*SSB_17_) versus in a bound but unwrapped state where a single domain of the *Ec*SSB tetramer binds ∼8 nt of ssDNA substrate (which we will denote as *Ec*SSB_8_).

### General two-step kinetic model for competitive binding dynamics

**Figure 2.**
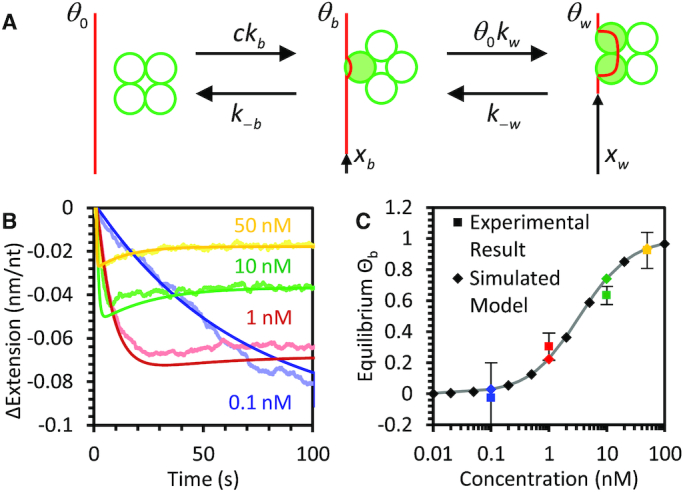
General model of two step binding and wrapping of ssDNA by *Ec*SSB. (**A**) The protein binds and wraps ssDNA in two distinct steps. First, *Ec*SSB bimolecularly binds to ssDNA in which the on rate is proportional to the protein concentration and dissociation rate is a constant. In the second step, bound protein interconverts between wrapped and unwrapped conformations. The wrapping rate is proportional to the fraction of protein-free ssDNA whereas the unwrapping rate is proportional to the fraction of ssDNA that is occupied by bound but unwrapped protein. The ssDNA extension reduction due to an unwrapped *Ec*SSB of the ssDNA is small but measurable, while the wrapped state significantly reduces the ssDNA extension. (**B**) Numerical simulation of the two-step binding model (smooth dark lines) reproduces the biphasic extension-time profiles that are consistent with experimental results (representative curves from Figure 1C replotted as light lines). Note, the simulated data are not individual fits to these experimental curves, but the numerical solution obtained from a single set of parameters determined from measured rates and amplitudes in multiple experiments. (**C**) The fraction of ssDNA-bound *Ec*SSB in the unwrapped state (*Θ*_b_) upon reaching equilibrium is predicted by the model (diamonds) and follows the shape of a standard binding isotherm (gray line) where the two states are equally occupied at 3 nM *Ec*SSB. These results agree with experimental data (squares), in which the equilibrium ssDNA extension change is converted into values of *Θ*_b_ and *Θ*_w_ using the corresponding reductions in ssDNA extension due to each state, *X*_b_ and *X*_w_. The solid line represents a fit to the free-energy dependence of the binding fractions as derived in the supplemental information.

To fully quantify our experimental results by connecting the ssDNA extension changes observed with specific *Ec*SSB wrap states and transitions between these states, we first need to establish a basic model. We start with a diagram of a generic two state binding model with minimal assumptions (Figure 2A):

(1) $$\begin{equation*}\begin{array}{@{}l@{}} {\Theta _0}\underset{{{k_{ - b}}}}{\overset{{{k_b}[SSB]}}{\rightleftharpoons}}{\Theta _b}\underset{{{k_{ - w}}}}{\overset{{{\Theta _0}{k_w}}}{\rightleftharpoons}}{\Theta _w}\\ \end{array}\end{equation*}$$

Here, Θ_0_, Θ_b_ and Θ_w_ are the fractions of ssDNA substrate that are protein free, occupied by bound (but not wrapped) *Ec*SSB_8_, and occupied by wrapped *Ec*SSB_17_, respectively. Though at 12 pN, we refer specifically to *Ec*SSB_17_ as the wrapped state, more steps could be added to the right side of this reaction diagram to represent higher order wrapped states accessible at lower forces (though such a complex system would be challenging to interpret analytically). *k*_b_ and *k*_-b_ are the bimolecular association and dissociation rates, respectively. *k*_w_ and *k*_-w_ are effective wrapping and unwrapping rates of a single *Ec*SSB, respectively. The only explicit assumptions made are that the initial rate of free protein binding is directly proportional to free protein concentration and that protein requires some additional bare ssDNA substrate to transition from the bound to the wrapped state. Additionally, a single bound or wrapped *Ec*SSB reduces ssDNA extension by a value of Δ*x*_b_ or Δ*x_w_*, respectively (both with units of nm, summing all bound proteins over the substrate returns the normalized extension change Δ*X* in units of nm/nt).

The rate at which proteins transition between these states can be written as a series of differential equations, similar to previously analyzed multistate models (44), as shown in detail in the supplemental information. This in turn allows us to numerically solve for the fraction of protein in each state over time for any given set of parameters. Due to the high number of free parameters in this model, fitting it to individual curves from single experiments can yield non-unique solutions. Rather, after empirical determination of the fundamental parameters (derived in the following sections and summarized in Table 1), we numerically solve the model with a unique set of parameters, which accurately reproduce the concentration-dependent biphasic binding profiles we observe experimentally (Figure 2B). Furthermore, the model accurately predicts the equilibrium balance of *Ec*SSB in either the bound or wrapped states over a wide range of *Ec*SSB concentrations, with the two states equally occupied at a critical concentration of ∼4 nM (Figure 2C). While *Ec*SSB preferentially wraps the ssDNA at low protein concentrations, the wrapped state becomes less stable as the concentration is increased.

**Table 1.** Parameters for two step binding model. Derived values for *Ec*SSB binding and wrapping at 12 pN, including rates at which the ssDNA–*Ec*SSB complex moves between states, average ssDNA contraction associated with each state, and the critical concentration at which the two states are equally occupied, are listed here

Parameters for two step binding model	
**Transition rates**	**(s** **^−1^** **)**
*k*_b_ (1 nM)	0.150 ± 0.025
*k*_*-*b_	0.0171 ± 0.0027
*k*_w_	1.40 ± 0.42
*k*_*-*w_	<0.01
*k* ^s^_*-*b_	0.113 ± 0.024
*k* ^s^_*-*w_	0.095 ± 0.022
**ssDNA compaction**	**(nm/nt)**
*Θ_b_*	−0.0159 ± 0.0052
*Θ_w_*	−0.083 ± 0.011
**Critical concentration**	**(nM)**
Simulation	3.40
Experiment	4.8 ± 1.8

One additional complication for proteins with finite binding site sizes filling a binding template is that the proteins could be inefficiently distributed, limiting saturation as detailed in the McGhee-von Hippel model (45,46). When there is a small length of ssDNA between two neighboring proteins, such that an additional protein will not fit, these regions of ssDNA will remain free of protein, even at saturating protein conditions. However, due to *Ec*SSB’s ability to diffuse quickly along the ssDNA substrates (32), we assume that proteins can reorganize after binding to maximize ssDNA saturation, such that both Θ_b_, and Θ_w_ are potentially able to approach 100%.

### Concentration-dependent interconversion of *Ec*SSB states

**Figure 3.**
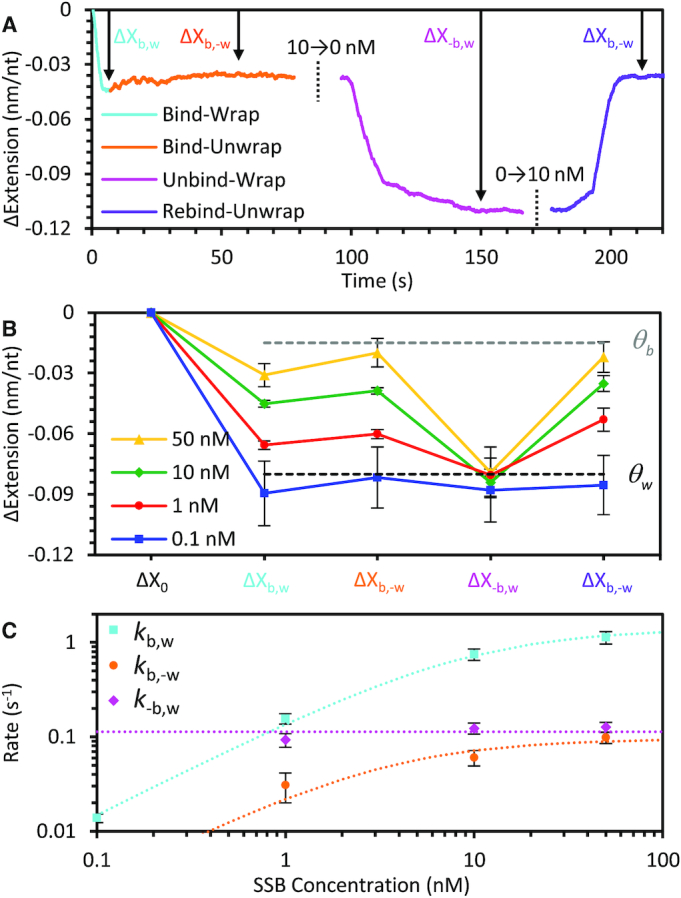
Concentration dependence of *Ec*SSB–ssDNA binding and wrapping at 12 pN template tension. (**A**) In the presence of free *Ec*SSB, the ssDNA extension first decreases due to the binding and subsequent wrapping of ssDNA by *Ec*SSB (bind-wrap, cyan). A maximum extension decrease of Δ*X*_b,w_ is reached before the *Ec*SSB unwrapping outpaces wrapping due to continued binding (bind-unwrap, orange), and the ssDNA extension change reaches a stable equilibrium of Δ*X*_b,-w_. Removal of free *Ec*SSB from solution results in some dissociation events without replacement; however, concomitant further wrapping of the bound *Ec*SSB results in a net extension-decrease of Δ*X*_-b,w_ (unbind-wrap, magenta). Reintroducing free protein allows *Ec*SSB to rebind to the *Ec*SSB–ssDNA complex that stimulates unwrapping events. This process registers as a net increase of Δ*X*_b,-w_ in the complex extension (rebind-unwrap, violet) (**B**) The net changes in extension of the *Ec*SSB–ssDNA complex after each step (data points with error bars) are averaged over multiple experiments for *Ec*SSB concentrations ranging from 0.1 to 50 nM. Connecting lines are guides to the eye showing how the down-up-down-up pattern seen in panel A is most pronounced at high concentration. As the *Ec*SSB concentration is increased, the change in extension during incubation is decreased. Removal of free *Ec*SSB results in a consistent ∼0.08 nm/nt reduction in ssDNA extension (dotted line), regardless of initial *Ec*SSB concentration. The net extension change is consistent with the previously observed length change associated with a single *Ec*SSB_17_ on a 70 nt ssDNA substrate (27). Upon reintroducing free *Ec*SSB, the complex's extension consistently reaches the same value as during the first incubation. (**C**) The average rate associated with each step of the *Ec*SSB–ssDNA interaction varies with free *Ec*SSB concentration. The bind-wrap step (cyan) is rate limited by the initial binding of *Ec*SSB from solution at low concentrations, resulting in a linear dependence, and reaches an asymptote at high *Ec*SSB concentrations. The bind-unwrap step (orange) is rate limited by the unwrapping events of *Ec*SSB, at a rate proportional to the bound but unwrapped *Ec*SSB fraction. The unbind-wrap step (magenta) occurs at a constant rate of *k*_-b,w_ = 0.11 s^−1^ and is independent of the initial *Ec*SSB concentration (see Supplemental information for detailed derivation).

To quantify the interconversion dynamics of *Ec*SSB, we measured the amplitude of extension change (Δ*X*) associated with each phase of our binding experiments (Figure 3A). Each phase is defined by the primary processes responsible for the observed change in the ssDNA extension. First, when *Ec*SSB is initially introduced, the ssDNA shortens as individual *Ec*SSB proteins bind then wrap the ssDNA (Figure 3A cyan, Δ*X*_b,w_), which we denote as the bind-wrap transition. Eventually, no more bare ssDNA is present (the substrate is saturated), preventing further compaction. However, at high protein concentrations, further additional protein can continue to bind if already-bound proteins unwrap and release some of the ssDNA substrate. This results in the second transition, where the ssDNA elongates, which we define as the bind-unwrap transition (orange, Δ*X*_b,-w_). The ssDNA eventually reaches an equilibrium state, with reduced net compaction, where more proteins are bound than can be accommodated if they were all in the wrapped state (which we refer to as oversaturated). Third, with free protein removed from the solution, no further binding can occur, but some *Ec*SSB dissociates into solution allowing further wrapping of bound *Ec*SSB, resulting in ssDNA shortening (magenta, Δ*X*_-b,w_), and this process is referred to as the unbind-wrap transition. Finally, reintroducing free protein once again elongates ssDNA by forcing *Ec*SSB to unwrap to accommodate more protein (violet, Δ*X*_b,-w_). Interestingly, while the final equilibrium extension is equal for both the first and second protein incubation, the transition is much faster when protein is reintroduced. We average the results from three or more independent experiments for each *Ec*SSB concentration (Figure 3B), showing several significant trends. The amount of ssDNA compaction (and underlying *Ec*SSB wrapping) decreases with increasing *Ec*SSB concentration. However, regardless of the initial *Ec*SSB concentration, the subsequent ssDNA extension upon removal of free protein (Δ*X*_-b,w_) converges at ∼0.08 nm/nt, which is the same as the net extension change observed with single-phase binding at [*Ec*SSB] ≤0.1 nM (Figure 1C, blue line). Thus, when free *Ec*SSB is scarce, the *Ec*SSB–ssDNA complex reproducibly returns to the same stable equilibrium state, in which excess *Ec*SSB dissociates (for which the substrate does not have sufficient length to wrap), and the rest remains stably wrapped on the observation timescale of 100 s. Thus, we designate this net extension as the characteristic extension change associated with the wrapped state (Θ_w_). This result compares well to the single molecule experiments at similar tensions (27), and we calculate that a ∼2 nm ssDNA compaction per *Ec*SSB tetramer averaged out over a long substrate with each protein occupying a binding site of ∼17 nt would result in a total ssDNA extension change of ∼0.1 nm/nt. In contrast, at the highest measured *Ec*SSB concentration, the wrapped state is destabilized, and the *Ec*SSB–ssDNA complex exhibits a small, but non-zero extension change. We therefore associate this ∼0.02 nm/nt extension change with the bound but unwrapped state of *Ec*SSB, which we further support with additional experiments detailed below.

### Binding and wrapping kinetics of *Ec*SSB

In order to measure the fundamental kinetic rates associated with *Ec*SSB dynamics, we fit the extension change over time for each phase of the binding experiment with a single-rate exponential function (sample fits shown in Supplementary Figure S3). These apparent rates are related to (but not exactly equal to) the fundamental rates of protein (un)binding and (un)wrapping, as defined by our model (see Supplemental Information). First, during the bind-wrap phase, extension decreases as *Ec*SSB binds from solution and then wraps the ssDNA. Thus, the measured rate (*k*_b,w_) depends on both the rates of initial bimolecular binding (*c·k*_b_) and wrapping (*k*_w_). At low protein concentrations, *k*_b,w_ increases linearly with *Ec*SSB concentration *c*, yielding a bi-molecular rate of protein binding to bare ssDNA of *k*_b_*=* 0.18 nM^−1.^s^−1^ (close to the diffusion limit for free *Ec*SSB), while at higher protein concentrations it saturates at a constant value corresponding to the fundamental wrapping rate, *k*_w_ = 1.8 s^−1^ (cyan line, Figure 3C, see Supplemental Information for derivation). Second, during the bind-unwrap phase, ssDNA extension starts to increase as a consequence of unwrapping events of bound-*Ec*SSB, which allows further protein binding from the solution. This rate increases with free protein concentration but is an order of magnitude slower than the rate of free protein binding bare ssDNA (*k*_b,-w_ *<< c·k*_b_). Therefore, the bind-unwrap process must be rate limited by the unwrapping events of already bound *Ec*SSB that release one OB-fold domain freeing up ssDNA substrate for additional protein binding. The high concentration asymptote of *k*_*-*w_= 0.10 s^−1^ is thus the fundamental rate of unwrapping at 12 pN in this solution condition (orange line, Figure 3C). Third, during the unbind-wrap phase the extension decreases as some *Ec*SSB dissociates, allowing other tetramers to wrap. Again, this process is much slower than the derived rate of wrapping (*k*_-__b,w_ *<< k*_w_), indicating this process is rate-limited by *Ec*SSB dissociation. Since this phase occurs in protein-free buffer, the effective dissociation rate *k*_-__b_ = 0.1 s^−1^ is independent of the initial *Ec*SSB concentration during incubation (magenta line, Figure 3C). Importantly, the above measured rates of unwrapping and dissociation are measured for protein saturated conditions. At lower free protein concentrations, and on a non-saturated ssDNA substrate, observed unwrapping and dissociation rates are much lower, consistent with the stable binding both observed here and in previous studies. Thus, as we will show in more detail below, the rates of *Ec*SSB unwrapping and dissociation are not constant but can be ‘stimulated’ by interprotein interactions.

### Force dependence of *Ec*SSB–ssDNA binding dynamics

**Figure 4.**
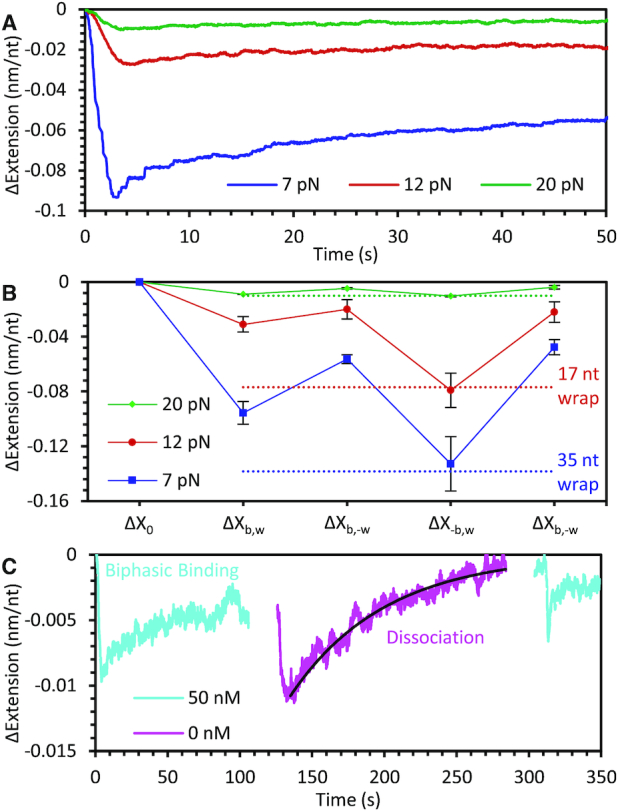
Force dependence of *Ec*SSB–ssDNA binding and wrapping. (**A**) As the force on the ssDNA template is decreased, the binding of 50 nM SSB causes a larger change in extension, consistent with *Ec*SSB accessing more wrapped states while bound to the ssDNA. A biphasic binding profile is seen at each force. (**B**) Average extension decrease at each phase of *Ec*SSB binding (compare to Figure 3B) is shown for each force. After removing free protein (ΔX_-b,w_), the average extension decrease at 12 and 7 pN is consistent with an *Ec*SSB tetramer in the 17 nt wrapped state (red dotted line) and in the 35 nt wrapped state (blue dotted line), respectively, every 70 nt along the ssDNA substrate. (**C**) At 20 pN applied force, *Ec*SSB wrapping is unstable and most *Ec*SSB is in an unwrapped state. After removing free protein (magenta line), *Ec*SSB will dissociate from the ssDNA without replacement, leaving bare ssDNA. The ssDNA’s extension return to its original value is fit with an exponential (black line) to measure the rate of *Ec*SSB dissociation. Biphasic binding after the reintroduction of *Ec*SSB (second blue line), indicates the ssDNA is mostly free of protein after the dissociation step.

Whereas we specifically detailed above *Ec*SSB wrapping dynamics while a force of 12 pN was maintained on the DNA substrate, these results are generalizable to other ssDNA tensions. We repeated the competitive binding measurements using 50 nM *Ec*SSB and observed biphasic binding at both lower (7 pN) and higher (20 pN) forces (Figure 4A). The measured extension change increases with decreasing force, indicating that higher order wrapped states become progressively stable as the template tension is lowered. At 7 pN (blue line, Figure 4B), the maximal extension change is observed after removing free protein to allow for increased wrapping (Δ*X*_-b,w_ = 0.13 nm/nt). Based on our low concentration data (Supplementary Figure S2), which resolves an average compaction event of ∼5 nm, this is consistent with the remaining protein occupying the *Ec*SSB_35_ state (5 nm/35 nt = ∼0.14 nm/nt). This is again consistent with previous single molecule experiments showing the *Ec*SSB_35_ mode favored at 7 pN tension (27). In contrast, the equilibrium complex extension change before the 50 nM *Ec*SSB is removed from solution is much smaller, indicating that at 7 pN *Ec*SSB_17_ becomes favored over *Ec*SSB_35_. This is similar to how *Ec*SSB_35_ can become favored over *Ec*SSB_65_ at high protein concentration in the absence of ssDNA tension. At 20 pN (green line, Figure 4B), wrapping is greatly destabilized, and most of the bound protein is unable to wrap, as evidenced by the minimal ssDNA compaction. Additionally, once free protein is removed, we measure a gradual extension increase over a ∼100 s timescale (Figure 4C). The final extension approaches the extension of the protein-free ssDNA, indicating complete dissociation of *Ec*SSB. This is also supported by the observation that as the protein solution is re-introduced there is a biphasic binding profile (bind-wrap followed by bind-unwrap) that typically occurs during the initial protein incubation with protein-free ssDNA. Fitting an exponential rate to this process returns a much slower rate of dissociation *k*_-__b_ = 0.017 s^−1^ with respect to the rate observed during the unbind-wrap transitions (0.1 s^−1^). Furthermore, we conducted force-jump experiments to test whether *Ec*SSB can remain bound to ssDNA at even higher tensions (Supplementary Figure S4). Here, first a stably wrapped *Ec*SSB–ssDNA complex is produced at 12 pN by incubating ssDNA with 50 nM *Ec*SSB and then removing the free protein from the solution. The ssDNA is then abruptly (<1 s) stretched until a tension of 60 pN is obtained and held for 10 s, before bringing the tension back down to 12 pN. The ssDNA equilibrates to an extension slightly longer than that prior to the force-jump but remains significantly lower than that of a protein-free ssDNA. Thus, while some *Ec*SSB dissociates during the force-jump, most remains bound and can rewrap when the ssDNA tension is brought back to 12 pN. This interpretation is further supported by a net increase in extension when protein is added back into the sample, indicating *Ec*SSB unwrapping events that are only observed on an *Ec*SSB-saturated ssDNA. We estimate the rate of protein dissociation (*k*_-b_) during the force jump by comparing the net ssDNA compaction due to wrapping just before and after the force-jump (*k*_-b_ = 0.017 s^−1^), which is consistent with the directly observed rate of *Ec*SSB dissociation at 20 pN (Supplementary Figure S4 inset). Therefore, the direct dissociation rate of unwrapped protein, *k*_-b_, is very weakly force dependent, though a minimum force is required to prevent wrapping and allow dissociation. This result supports the hypothesis that in the *Ec*SSB_8_ mode only a single OB-fold domain on the *Ec*SSB tetramer is bound to ssDNA, resulting in minimal ssDNA compaction, such that this unwrapped binding mode does not require ssDNA to assume a specific conformation that could be prohibited by substrate tension. In contrast, the various higher order wrapping modes in which *Ec*SSB greatly compacts ssDNA are therefore strongly destabilized by applied force on the ssDNA substrate.

In contrast to the above experiments, which are performed with the ssDNA substrate under tension, the majority of previous experiments performed in the absence of force have not observed *Ec*SSB binding in this unwrapped state. There are exceptions, however, for short ssDNA substrates that cannot accommodate wrapped protein, including tetramers simultaneously binding several 8 nt long oligos and transiently binding short ssDNA overhangs before unraveling a hairpin (29,30). We have devised one additional experiment that forces *Ec*SSB to bind ssDNA in an unwrapped state in the absence of force. While AFM imaging cannot directly resolve a large protein binding to a small ssDNA oligo, a dsDNA marker can be used to visualize ssDNA binding (39). Specifically, we incubate *Ec*SSB with a 100 bp dsDNA construct with an 8 nt poly(dT) ssDNA overhang at equimolar concentration (100 nM during incubation diluted to 5 nM for deposition) in a buffer containing 10 mM Na^+^, 10 mM Mg^2+^, 10 mM HEPES, pH 7.5. Previous AFM experiments using the same concentration of Mg^2+^ have confirmed that *Ec*SSB specifically binds the ssDNA region of these hybrid constructs (47). The *Ec*SSB does not bind dsDNA, and the ssDNA segment is too short to be wrapped (<17 nt). But when the sample is deposited on an aminopropyltriethoxy silane (APS) coated mica surface and imaged using AFM, we observe colocalization of *Ec*SSB with one terminus of the dsDNA region, where the ssDNA overhang is located (Supplementary Figure S5). It is possible that after binding, *Ec*SSB could partially melt the dsDNA at the ssDNA junction in order to create a longer substrate to stabilize wrapping. However, the fact that *Ec*SSB does not generally melt fully dsDNA constructs and that we do not observe binding to the blunt end dsDNA of this construct indicates that the 8 nt ssDNA overhang is sufficient to enable initial binding. These results provide further validation that *Ec*SSB is able to bind ssDNA without wrapping. Unfortunately, AFM imaging requires particular non-physiological salt conditions and DNA or protein sticking to the surface can potentially interfere with binding, so a direct calculation of *K*_d_ based on the number of DNA substrates bound by protein is not generalizable. The fact that most substrates are unbound, however, is consistent with greatly reduced binding affinity as compared to our experiments with a long ssDNA substrate where 100 pM protein saturates the substrate. Thus, as the length of the ssDNA template is increased, *Ec*SSB will transition to a more stable wrapped state, such that this unwrapped state is not generally observed on longer substrates in the absence of substrate tension.

### Mutant *Ec*SSB experiments confirm role of *Ec*SSB tetramerization in binding and wrapping

**Figure 5.**
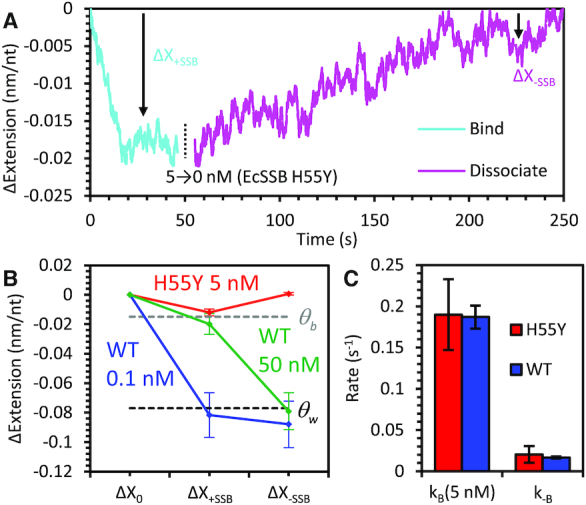
*Ec*SSB mutant exhibits modified binding and wrapping behavior. (**A**) An ssDNA molecule held at 12 pN is incubated with 5 nM non-tetramerizing *Ec*SSB mutant H55Y. Compared to WT binding and wrapping at comparable concentration, the initial decrease in ssDNA extension during *Ec*SSB H55Y binding (cyan) has a much smaller amplitude with no secondary increase of extension. Upon the removal of free *Ec*SSB H55Y (magenta), the unbind-wrap process seen with the WT is also not observed. Instead the ssDNA extension slowly increases indicating direct dissociation events. Both the binding and dissociation curves are fit to single exponential functions to calculate binding and dissociation rates. (**B**) Average ssDNA extension changes after *Ec*SSB H55Y binding and dissociation. The ssDNA extension while bound by monomeric H55Y is consistent with that of the predicted bound but unwrapped *Ec*SSB_8_ state (Figure 3B). After dissociation, the ssDNA extension approaches its initial value, indicating dissociation of *Ec*SSB H55Y. (**C**) Rates of *Ec*SSB H55Y binding and dissociation. The rate of binding (*k*_B_) for 5 nM (monomer concentration) *Ec*SSB H55Y is the same as the rate of initial binding of an equivalent concentration of WT *Ec*SSB (1.25 nM tetramer concentration). The rate of H55Y dissociation is the same as the direct dissociation rate of WT *Ec*SSB at forces >20 pN that inhibit wrapping.

To support our above interpretation of the collective *Ec*SSB binding and wrapping dynamics, we utilize a previously characterized *Ec*SSB mutant with the histidine at residue 55 replaced with tyrosine (H55Y) (48,49), which does not form tetramers at low protein concentrations (Supplementary Figure S6). Since the wrapped states of *Ec*SSB require ssDNA association with multiple OB-fold domains of the tetramer, monomeric *Ec*SSB is unable to wrap ssDNA. Incubating the ssDNA held at 12 pN of tension with 5 nM monomeric H55Y mutant yields a single-phase binding profile with a net extension change of ∼0.015 nm/nt (Figure 5A). This extension change is consistent with the equilibrium extension change that is observed with high concentrations of wild type (WT) *Ec*SSB (Figure 5B). In the absence of free protein, H55Y dissociates from the ssDNA with a rate that is consistent with the direct dissociation observed with WT *Ec*SSB at higher forces (Figure 5C). Moreover, the bimolecular on rate *k*_B_ of the monomeric H55Y at 5 nM agrees with the equivalent monomer concentration of the WT tetramer (1.25 nM), supporting our hypothesis that the initial binding of the ssDNA substrate (before wrapping) is a simple diffusion limited process that only requires binding to a single OB-fold domain. Similarly, the rate at which the monomeric *Ec*SSB H55Y dissociates from ssDNA (*k*_-B_) is consistent with the rate of dissociation of WT *Ec*SSB when enough force is applied to destabilize wrapping. Agreement between these two pairs of rates also indicates that the binding affinity for this mutant (*K*_d_*= k*_-b_*/k*_b_*≈* 0.1 nM) is the same as that of single domain binding of WT *Ec*SSB. These results support our model, in which *Ec*SSB binding ssDNA without wrapping exists as a transition state through which the complex must pass before a free protein can assume a wrapped conformation and before a wrapped protein can fully dissociate from the ssDNA. Also, since monomeric *Ec*SSB barely compacts ssDNA, its binding has only a weak force dependence, and should behave similarly with other experimental techniques that do not apply ssDNA substrate tension. In contrast, its binding mode (in which the ssDNA lies tangential to the protein rather than wrapping around it), is likely highly electrostatic in nature and can be efficiently screened in high salt buffers.

### Nearest neighbor interactions stimulate *Ec*SSB unwrapping and dissociation

**Figure 6.**
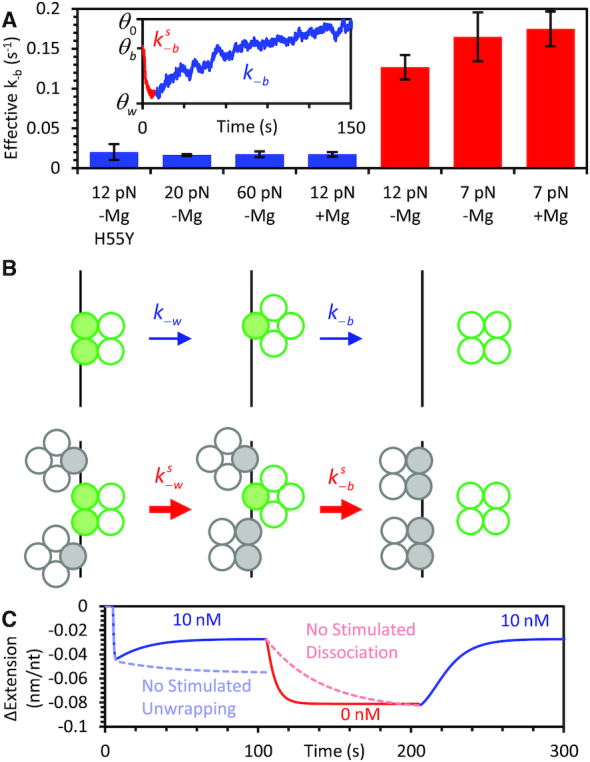
*Ec*SSB stimulated unwrapping and dissociation due to near neighbor effects. (**A**) Direct dissociation of *Ec*SSB is observed with the H55Y mutant due to compromised tetramer formation, with WT at forces >20 pN in the absence of Mg^2+^ or at forces >12 pN in the presence of 4 mM Mg^2+^. In either case the direct dissociation from an unsaturated ssDNA complex is measured to be ∼0.015 s^−1^ (blue bars). In contrast, *Ec*SSB dissociation from an oversaturated ssDNA complex that is concomitant with wrapping events of neighboring *Ec*SSB occurs 10-fold faster at a rate of ∼0.15 s^−1^ (red bars). Inset shows the unbind-wrap process at 20 pN in which rapid stimulated dissociation (*k*^s^_-b_) from the oversaturated complex (red) is followed by the slow dissociation (*k*_-b_) events from the now unsaturated complex (blue)). (**B**) Schematic (ssDNA in black and *Ec*SSB in green) showing near neighbor stimulation of dissociation and unwrapping events. The net interacting interfaces of *Ec*SSB–ssDNA decreases upon *Ec*SSB dissociation from an unsaturated ssDNA, leaving behind free ssDNA. However, in an oversaturated *Ec*SSB–ssDNA complex the net loss of protein-ssDNA interactions is minimal or none as near neighbors (gray circles) may compete to accommodate the substrate made available by unwrapping or dissociation events. (**C**) Model correction with simulated dissociation and unwrapping. The proposed two-step model (Figure 2) reproduces the observed experimental *Ec*SSB–ssDNA wrapping kinetics (Figure 1) in the presence and absence of free *Ec*SSB (blue and red lines) only when the stimulated dissociation and unwrapping is taken into account. Disregarding *k*_-b_ or *k*_-w_ stimulated dissociation and unwrapping (by keeping *k*_-b_ or *k*_-w_ constant) in the model results in a loss of the biphasic extension as seen with high *Ec*SSB concentrations (light blue dotted line), and a much slower unbind-wrap process (light red dotted line), which is inconsistent with the observed results (Figures 1D and 3A).

In our experiments, we measure two distinct rates of *Ec*SSB dissociation (Figure 6A). The dissociation observed from an *Ec*SSB oversaturated complex, which is concomitant with further wrapping of the bound *Ec*SSB (achieved by oversaturating the ssDNA with high concentration *Ec*SSB, and then removing free protein), is faster, and occurs on the timescale of 10 s. In contrast, when wrapping is inhibited by high forces (*F* > 15 pN) or when tetramerization (H55Y mutant) is inhibited, we observe much slower dissociation events (∼100 s) upon removing free protein from the solution. This slow dissociation rate of *Ec*SSB is that of a single protein in isolation, when the tetramer leaves a non-saturated ssDNA substrate by the release of its last bound OB-fold domain, leaving bare ssDNA behind. In contrast, the fast rate of dissociation is only observed when the ssDNA is oversaturated, when there are too many bound tetramers for each to wrap the ssDNA substrate and any ssDNA released by a dissociating protein is immediately bound by an OB-fold domain of its neighbor as it transitions to a wrapped state.

As a result, the dissociation of *Ec*SSB is ‘stimulated’ by interprotein interactions, specifically the presence of other bound but unwrapped proteins on the ssDNA substrate. This fast rate of stimulated dissociation can be potentially explained by the energetic favorability of the various states of *Ec*SSB wrapping. When an isolated *Ec*SSB tetramer unwraps and dissociates from an ssDNA substrate, each associated OB domain must come off in succession until it is entirely free of the ssDNA (top path, Figure 6B). Since multiple OB-fold domains are bound to the ssDNA (especially in the absence of substrate tension where higher order wrap states are enabled), there is a large energy barrier to simultaneously breaking all these bonds, resulting in extremely slow dissociation. In contrast, if the ssDNA is oversaturated with *Ec*SSB (bottom path, Figure 6B), any OB-fold domain that releases the ssDNA during an unwrapping or dissociation event is replaced with an OB-fold domain of a neighboring protein as it transitions to a more wrapped state. This coordinated replacement lowers the free energy barrier for both unwrapping and dissociation, greatly increasing their kinetics. A similar phenomenon, where the simultaneous binding of proteins from solution increases the rate of bound protein dissociation, has been observed in many other systems (50). The signature of facilitated protein dissociation, as discussed in (50), is its progressive enhancement proportional to increasing bulk protein concentration. In contrast, our experiments display an increased rate of dissociation even in the absence of free protein. However, the underlying effect is likely similar in both cases, in which dissociation of one protein attached to the DNA substrate at several sites is enhanced through substrate being gradually displaced by another protein, either coming from the bulk solution, or already bound to DNA.

We further show that both stimulated dissociation and stimulated unwrapping must be taken into account in order for the generalized two-step binding model to accurately reproduce our experimental data. In contrast, assuming *k*_-b_ and *k*_-w_ are constant and setting them to the values as determined in the absence of nearest neighbor interactions produces binding curves that lack two of the main features of our experimental data (Figure 6C). First, the lack of stimulated unwrapping eliminates the biphasic profile of the initial binding curve, as stable wrapping outpaces unwrapping at equilibrium. Second, the absence of stimulated dissociation results in a much slower unbind-wrap transition than what is observed. Even in the absence of free protein, *Ec*SSB wrapping is rate limited by the availability of free ssDNA which can only be produced through protein dissociation for a saturated substrate. Instead, the rates of both dissociation and unwrapping must effectively increase by an order of magnitude as the ssDNA substrate is oversaturated with *Ec*SSB.

### Progressively decreasing substrate length triggers *Ec*SSB nearest neighbor interactions and dissociation

**Figure 7.**
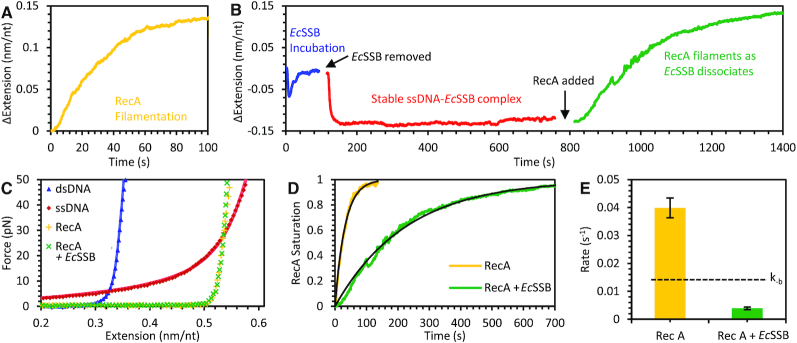
Dissociation of *Ec*SSB during RecA filament formation. (**A**) RecA filamentation (100 nM RecA with 100 μM ATPγS) on bare ssDNA at 7 pN occurs at a timescale of ∼10 s and is registered as an increase in ssDNA length due to the increase in its persistence length. (**B**) RecA filamentation on a maximally wrapped *Ec*SSB–ssDNA complex at 7 pN. The *Ec*SSB–ssDNA complex is obtained by first incubating the bare ssDNA at 7 pN with 50 nM *Ec*SSB and then removing the free *Ec*SSB from the solution as described in the text. Here, RecA forms filaments, presumably displacing *Ec*SSB from ssDNA, but at a much longer (∼100 s) timescale. (**C**) The resultant force-extension profiles of the RecA-ssDNA filaments formed in both (A) and (B) are identical, which confirms complete RecA filamentation in either case. (**D**) Normalized extension-time profiles of RecA filamentation kinetics on the bare ssDNA (yellow) and *Ec*SSB–ssDNA complex (green) yield simple exponential functions (black) (**E**) Comparison of the RecA filamentation rates in (A) and (B) shows that the RecA filamentation on the *Ec*SSB–ssDNA complex is rate limited by the *Ec*SSB dissociation rate, *k*_-b_ (dashed black line).

Our results demonstrate that an excess of free protein in solution leads to an oversaturated ssDNA substrate. Moreover, this oversaturation stimulates *Ec*SSB unwrapping events, favoring less wrapped *Ec*SSB states, in agreement with previous bulk solution observations (25). Alternatively to increasing free protein concentration, decreasing the length of an ssDNA substrate with *Ec*SSB already bound also increases local protein density. This process naturally occurs during lagging strand synthesis, in which the DNA polymerase advances along an ssDNA template, displacing SSBs. Such a direct interaction has been recently observed *in vitro* for polymerase-SSB pairs from other biological systems (51). However, SSB can be displaced by other proteins, such as experiments showing ATP driven ssDNA translocase actively pushing *Ec*SSB off the end of a short, free-ended ssDNA segment (52). We intended to observe how many *Ec*SSBs along a long ssDNA substrate can be removed through non-specific competition with proteins. To this end, we introduced RecA, which after nucleation events forms filaments, and displaces *Ec*SSB (53). Because RecA–ssDNA filamentation requires Mg^2+^ cations, we first investigated *Ec*SSB–ssDNA binding dynamics in a solution containing Mg^2+^ (50 mM Na^+^, 4 mM Mg^2+^, 10 mM HEPES, pH 7.5). The overall binding dynamics of *Ec*SSB remain similar in the Mg^2+^ buffer (Supplementary Figure S7). In the presence of Mg^2+^ the local secondary structures slightly shorten the ssDNA molecule at lower forces (38) (Supplementary Figure S7A). Correcting for this additional change in the ssDNA extension without protein yields the same equilibrium extension changes as observed in the Mg^2+^ free buffer (Supplementary Figure S7D). However, as the free protein in the solution is removed, the presence of Mg^2+^ resulted in *Ec*SSB dissociation at 12 pN, where after the unbind-wrap transition initially contracts the ssDNA further there is a long timescale increase in extension as protein is removed from the substrate (Supplementary Figure S7B). Interestingly, the measured dissociation rate *k*_-b_ at 12 pN in the Mg^2+^ buffer is consistent with the same rate measured at 20 pN in the Mg^2+^ free buffer, suggesting that fluctuations between the bound *Ec*SSB_8_ and wrapped states are enhanced in the presence of Mg^2+^, as expected at higher ionic strength. We did not observe dissociation, however, at 7 pN, even in the presence of Mg^2+^. For this reason, we investigated the displacement of the ssDNA-bound *Ec*SSB by RecA filamentation at 7 pN of applied tension in the Mg^2+^ buffer (Figure 7). First, we examined RecA filamentation on an *Ec*SSB-free ssDNA substrate. A Mg^2+^ buffer solution containing 100 nM RecA and 100 μM ATPγS (a slowly hydrolyzable ATP analog) was introduced to an ssDNA molecule held at 7 pN. The RecA–ssDNA nucleoprotein complex is formed via a slower nucleation step followed by a faster, irreversible directional filamentation (54–57). As the filamentation proceeded, the increase in the rigidity of the RecA–ssDNA complex was registered as a gradual increase in the ssDNA extension (Figure 7A). The extension over time was not linear, as would be the case for filaments growing from a fixed number of nucleation sites, but rather exponential, similar to an idealized array of binding sites becoming occupied at a set rate until reaching saturation. Next, we repeated this experiment on an *Ec*SSB–ssDNA complex. To do so, we first incubated the ssDNA molecule that was held at 7 pN with 50 nM *Ec*SSB in the Mg^2+^ buffer and subsequently rinsed out the free *Ec*SSB from solution (Figure 7B). After the unbind-wrap transition, the *Ec*SSB–ssDNA complex stably equilibrates in its maximally wrapped state (predominantly with *Ec*SSB_35_ at 7 pN) for long timescales (∼1000 s) with no significant dissociation observed. Then we incubated the *Ec*SSB–ssDNA complex with 100 nM RecA and 100 μM ATPγS as before, but RecA filamentation now requires a ∼10× longer timescale for full saturation compared to starting with *Ec*SSB-free ssDNA. The resulting protein-ssDNA complex after either procedure, however, is a completely RecA-filamented ssDNA, as evidenced by the same subsequent force-extension curves (58) (Figure 7C). This indicates that RecA filamentation resulted in complete dissociation of *Ec*SSB that otherwise was highly stable in its wrapped conformation. The total degree of RecA saturation can be calculated over the timescale of each experiment using the instantaneous ssDNA extension relative to the final extension (Figure 7D). Fitting rates to these curves yields the rate of RecA filamentation both along protein-free ssDNA and *Ec*SSB-wrapped ssDNA (Figure 7E). The fact that *Ec*SSB slows RecA filamentation by an order of magnitude indicates that the rate of *Ec*SSB dissociation must be the rate limiting step in this process. While prior to the introduction of RecA, the *Ec*SSB was stably bound for hundreds of seconds, progressively larger and more numerous RecA filaments reduce the amount of available ssDNA for *Ec*SSB to bind, stimulating unwrapping /dissociation events due to nearest neighbor interactions. Interestingly, the observed 0.003 s^−1^*Ec*SSB dissociation/RecA filamentation rate, is even slower than the 0.017 s^−1^ rate of *Ec*SSB dissociation that occurs at high enough ssDNA tensions to inhibit wrapping. The most likely reason is that initial RecA nucleation events are partially inhibited by the presence of *Ec*SSB. However, the fact that the extension over time curve retains its exponential like form indicates *Ec*SSB is dissociating across the entire substrate, not in a sequential manner directly in front of each growing RecA filament, which would result in a linear extension increase over time. Indeed, given the ability of *Ec*SSB to dissociate along the ssDNA template, the protein should be able to reorganize to allow the procession of RecA filaments regardless of the exact location of dissociation events.

## DISCUSSION

### Generalized model enables study of less defined biological systems

This study had two main objectives. First, by using a long ssDNA substrate able to bind hundreds of *Ec*SSB tetramers, we can expand on the results of previously published studies focused on the single molecule interactions (27,59). Second, using what was already known about *Ec*SSB, including its occupation of multiple different wrapped states as a function of force, we were able to validate a generalizable model that accurately represents protein dynamics (Figure 2). Furthermore, the methods detailed here allowed for the determination of such fundamental parameters as binding affinity and rates of interconversion between binding states. While *Ec*SSB is a model system, most other proteins that specifically bind ssDNA are less studied. Whether a protein exhibits such behavior as ssDNA wrapping, concentration dependent switching between binding conformations, and interprotein interactions that either stabilize binding or promote dissociation can be directly measured in comparison to *Ec*SSB. For example, the retrotransposon long interspersed nuclear element 1 (LINE1) encodes for a protein ORF1p, a homo-trimer that shares binding characteristics with *Ec*SSB (60). This model can be used to detail the wrapping dynamics of such proteins.

### Effects of ssDNA tension and conformation on *Ec*SSB kinetics

While our experiments necessarily apply force on the ssDNA substrate to measure extension and bias wrapping states, our measured kinetics can be related to the behavior of *Ec*SSB under physiological conditions. Since the binding of *Ec*SSB to ssDNA without wrapping results in minimal ssDNA compaction, the kinetics of initial *Ec*SSB binding are force insensitive. In fact, the bimolecular binding rate constant we measure (*k*_b_*=* 0.18 nM^−1^ s^−1^) is equal in magnitude to a previously measured value in stopped flow assays (33). Similarly, the final step of complete protein dissociation (the breaking of the last OB-fold domain-ssDNA interaction) should also be nearly force independent, though at lower forces dissociation is greatly slowed due to the stability of wrapping. However, electrostatic screening by higher salt concentrations in combination with applied force allows for even faster dissociation (61). In contrast, *Ec*SSB wrapping, which greatly compacts ssDNA, is highly force dependent. By applying a force of 12 pN, we observe a rate limiting wrapping step at *k*_w_= 1.8 s^−1^, while in the absence of force, wrapping immediately occurs after binding on a millisecond timescale (33). This fast wrapping step was resolved from initial bimolecular binding, however, using a laser temperature-jump assay (34). Thus, under physiological conditions *Ec*SSB will first loosely bind the disordered free ssDNA at a diffusion limited rate, then immediately wrap the ssDNA, assuming there is sufficient substrate length to accommodate the increased binding site size. Once all ssDNA is occupied by fully wrapped protein, all additional binding of *Ec*SSB must be coupled with partial unwrapping of already bound protein. This results in the biphasic extension profiles we observe, including a measured rate of unwrapping at high protein concentrations on a 10 s timescale. A similar phenomenon was previously observed on a short 70 nt long substrate, where the binding of a second protein occurs at a rate two orders of magnitude slower than the first, due to the necessity of the first bound protein to unwrap from the 65 to the 35 nt wrap state. There are likely different kinetic rates between all the different possible wrapped states, and observations of a single protein can be used to construct an energy landscape (27). Here we observe a system of many proteins, and though this necessarily does not allow for precise measurements of each individual protein, the ensemble behavior is analyzed to extract rates. By fitting these kinetics to a general multistate model, we can show definitively that interactions between *Ec*SSB must be able to stimulate both the unwrapping and dissociation of neighboring proteins.

### A competitive binding mechanism allows oversaturation and stimulates dissociation

We show that the ssDNA is oversaturated via stimulated unwrapping when the free protein is abundant in solution. Furthermore, we find that the critical protein concentration where the *Ec*SSB_17_ and *Ec*SSB_8_ states are equally occupied (∼4 nM, Figure 2C) is significantly higher than the equilibrium dissociation constant that we measure for *Ec*SSB_8_ binding to our long ssDNA without free ends (*K*_d_ = 0.1 nM). This observation strongly supports the idea that the weaker *Ec*SSB_8_ binding affinity and its faster dissociation from the oversaturated *Ec*SSB–ssDNA complex is a consequence of competitive displacement of ssDNA from the less-wrapped *Ec*SSB by its nearest-neighbor *Ec*SSB. Interestingly, previous single molecule force spectroscopy studies measured even faster *Ec*SSB dissociation at high ssDNA tensions, characterized by loss of fluorescent signal of labeled proteins on a 70 nt ssDNA segment (27,59). These experiments differ from ours in two key aspects. First, we use a very long ssDNA substrate (8.1 knt), such that even with the ability to slide, the vast majority of bound proteins will never reach the end of the complex. In contrast, the 70 nt single protein binding sites are flanked by dsDNA junctions, with which a bound protein will be in constant contact. Second, there is the possibility that the presence of many bound proteins stabilizes the complex. That is, while proteins unable to wrap quickly and irreversibly dissociate when free protein is removed, the remaining wrapped proteins remain bound and at least partially wrapped such that we do not observe any change in ssDNA extension over hundreds of seconds even at 12 pN ssDNA tension (at least in the absence of Mg^2+^). This would make the timescale of full dissociation at least an order of magnitude slower than the timescale for even 100 pM protein to saturate the ssDNA, implying a sub-10 pM dissociation constant. However, we were not able to measure saturated binding of ssDNA by such low protein concentrations, though this also could be a result of protein instability/precipitation when so diluted in our experimental buffer at room temperature over the timescale of ∼1 h (Supplementary Figure S1). This discrepancy could reasonably be explained by previous arguments that *Ec*SSB has a degree of cooperative binding behavior, based on AFM images (62) and electrophoretic mobility shift assays (18), which showed *Ec*SSB mixed with plasmid DNA tended to form either fully saturated or bare DNA complexes rather than intermediates. If this cooperative effect is also present in our experiments, it could result in a higher binding affinity of *Ec*SSB on a long, saturated substrate as compared to a single protein on a short substrate.

### Stimulated unwrapping and dissociation are near isoenergetic processes

We show that the kinetics and equilibrium of *Ec*SSB binding to ‘unsaturated’ vs ‘oversaturated’ ssDNA differ dramatically, resulting in stimulated dissociation and stimulated unwrapping events only from the oversaturated state. The oversaturated complex occurs when more than one *Ec*SSB tetramer is bound per binding site (20-60 nt, depending on force, Supplementary Figure S1 and S2). Thus we hypothesize that the two ssDNA-bound *Ec*SSB tetramers are no longer influenced by each other once the average distance separating them becomes larger than the typical length (*L*) that the *Ec*SSB can diffuse on ssDNA during the time ∼10 s of its stimulated dissociation, which can be estimated to be (*L ≥ D*^.^*t*)^0.5^∼ (300 nt^2^/s*^.^*10 s)^0.5^) ∼ 55 nt, where D is the *Ec*SSB diffusion coefficient on ssDNA (32). While this is a rough estimate, it yields a plausible explanation for the *Ec*SSB density on ssDNA that separates its unsaturated and oversaturated binding regimes.

Interestingly, we find that the stimulated dissociation rate, *k^s^_-b_* is close in magnitude to the *Ec*SSB_17_ stimulated unwrapping rate, *k*^s^_-w_ ∼ 0.10 s^−1^, suggesting that these two rates might be limited by the timescale for ssDNA to peel from a single *Ec*SSB OB-fold domain. Simultaneous rebinding of this released ssDNA by another neighboring *Ec*SSB stimulates the dissociation of an *Ec*SSB_8_ by an order of magnitude. This is an almost isoenergetic process that does not require complete OB–ssDNA dissociation, but instead allows gradual replacement of one OB-fold domain for another. We suggest that the mechanism of stimulated dissociation from its oversaturated ssDNA complex is similar to the previously established mechanism of rapid diffusion of tightly wound *Ec*SSB on bare ssDNA (32). *Ec*SSB diffusion on ssDNA was shown to proceed via a reptational mechanism without protein unwrapping or dissociation. This process was shown to be much faster than the ssDNA wrapping dynamics, as it does not involve releasing all the OB-fold domains from ssDNA. Instead only small regions (2–5 nts) of ssDNA are temporarily released and immediately replaced by adjacent nts, leading to small bulges moving over *Ec*SSB, allowing for fast diffusion (61). This process results in much faster *Ec*SSB motion on ssDNA, while remaining fully bound and wrapped. Similarly, the stimulated *Ec*SSB dissociation and unwrapping on an oversaturated ssDNA complex is much faster than from an unsaturated ssDNA, as the *Ec*SSB–ssDNA interactions of one *Ec*SSB are being gradually replaced by similar interactions with its nearest neighbor. This interpretation is consistent with previous experiments showing that labeled *Ec*SSB on a ssDNA substrate could be rapidly exchanged with free unlabeled *Ec*SSB (35). If such an exchange required the complete dissociation of the bound protein before replacement, this would result in a large energetic barrier and slow kinetics. Instead, the incoming protein must partially bind the substrate as the outgoing protein peels off. A similar process was also detailed where an *Ec*SSB tetramer can directly transfer between two distinct ssDNA molecules (36). Again, for such a process to be energetically feasible, the tetramer cannot fully release one substrate before binding the next, but instead individual OB-fold domains can sequentially transfer from one substrate to the next, reducing the energy barrier to transfer. Thus, the same underlying process results in both excess of *Ec*SSB, allowing a substrate to rapidly exchange proteins, and an excess of ssDNA binding sites, allowing a protein to rapidly exchange substrates.

### Rapid *Ec*SSB kinetics ensures maximum ssDNA coverage during genomic maintenance

The rates at which ssDNA regions are produced, bound by SSBs, and eventually replicated into dsDNA are regulated by the ongoing coordinated enzymatic activity of the DNA polymerases, helicases, RecA, etc., during genomic maintenance processes. Extensive studies of *Ec*SSB with both traditional biochemical bulk assays and single molecule approaches have shown that *Ec*SSB is not easily removed from ssDNA substrates. While this feature is necessary to protect transiently formed ssDNA, it raises the question as to how exactly *Ec*SSB tetramers that are wrapped on ssDNA undergo rapid complex rearrangements including protein dissociation and re-association that are required to keep up with the enzymes involved in genomic maintenance. Furthermore, if *Ec*SSB is able to bind short ssDNA segments through a single OB-fold domain, as this work shows in agreement with limited previous observations, we must also ask why this conformation is not readily observed in most experiments.

As the amount of *Ec*SSB in bacteria is kept at the level sufficient for saturation of all available ssDNA (63–65), there should be a constant exchange of the ssDNA substrate within the saturated complex with *Ec*SSB. As most ssDNA is always *Ec*SSB-saturated, the mechanism of rapid *Ec*SSB diffusion on bare ssDNA (32) likely does not contribute significantly to the rapid *Ec*SSB turnover. Also, a massive *Ec*SSB transfer over long distances between the saturated and bare ssDNA is unlikely to be the main mechanism of such *Ec*SSB turnover, as it requires these distant ssDNA regions to be in direct contact with each other. Our results suggest novel pathways of the efficient *Ec*SSB exchange between distant ssDNA regions, which involve fast *Ec*SSB dissociation into bulk solution followed by the fast re-association with newly generated bare ssDNA. Such a process would allow for rapid *Ec*SSB recycling while maintaining complete ssDNA protection, as suggested by a recent single molecule study (66).

**Figure 8.**
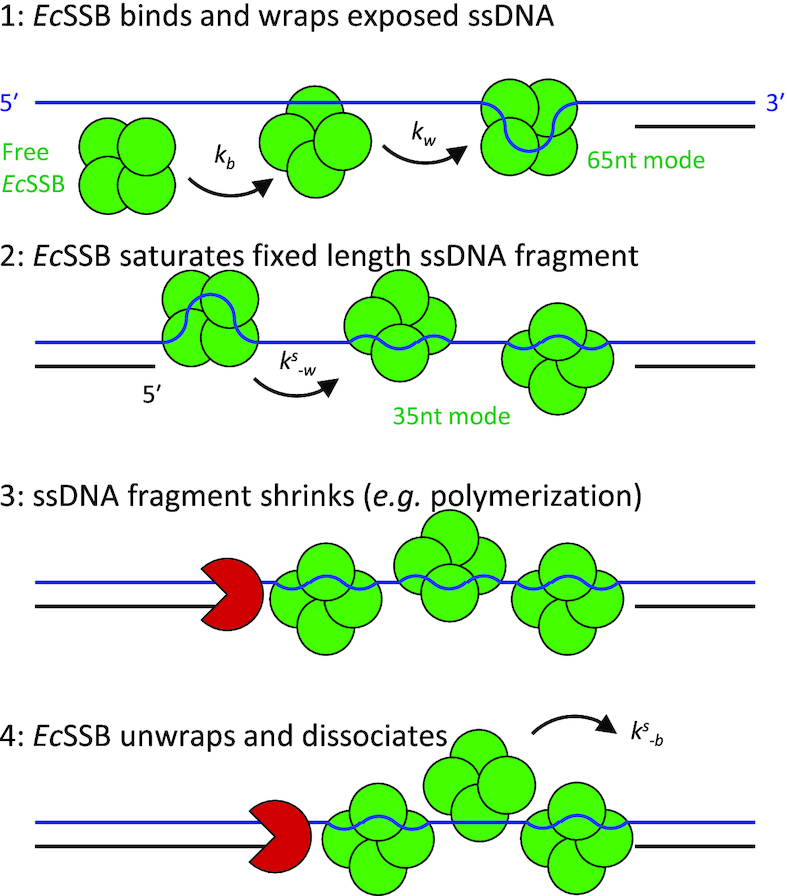
Self-regulation of protein density mechanism. (1) As an ssDNA region is gradually exposed, free *Ec*SSB immediately binds the substrate at a diffusion limited rate before immediately wrapping in the 65 conformation. (2) As more proteins continue to bind, interprotein interactions stimulate partial unwrapping into the 35 state until the ssDNA segment is saturated. (3) As the ssDNA segment shrinks in length, due to polymerization for example, the substrate can no longer accommodate all currently bound proteins. (4) Stimulated unwrapping followed by stimulated irreversible dissociation allows for rapid regulation of protein density on the ssDNA. This process can happen along the entire *Ec*SSB–ssDNA complex length, simultaneously dissociating many *Ec*SSB tetramers as needed and thereby self-regulating its density. This mechanism ensures fast turnover of ssDNA substrate to the processing enzyme as it translocates along the ssDNA template, while providing maximal ssDNA coverage at any given time.

Taken together, the results described in this study provide mechanisms to regulate the density of the ssDNA-bound *Ec*SSB, which is central for its transient role during genome maintenance and replication. Based on the findings in this study, we propose a mechanism of self-regulation of protein density, a phenomenon that emerges directly from competitive *E*cSSB binding dynamics (Figure 8). *Ec*SSB protein immediately binds any free nucleotides in transiently formed ssDNA regions, such as by the advancing of a helicase. In contrast to dissociation and unwrapping, our analysis shows that the processes of binding and wrapping are very fast and act as simple bimolecular processes. Additional protein binding to an ssDNA template of finite length, such as an Okazaki fragment, will promote partial unwrapping of *Ec*SSB_65_, such that the substrate is saturated with *Ec*SSB in an intermediately wrapped state such as *Ec*SSB_35_. This was previously seen with the human mitochondrial SSB that is structurally and functionally similar to *Ec*SSB (67). However, we must also note that *Ec*SSB exists in a dynamic equilibrium between its distinct modes that are able to diffuse along the DNA without dissociation (31). Therefore, a processing enzyme, may displace the wrapped *Ec*SSB during synthesis by pushing it forward along the template strand. Such a scheme is supported by a recent study demonstrating species-specific inter-protein interactions between DNA polymerase and SSB that enhance replication rates (51). One possibility is that the advancing polymerase actively kicks off each *Ec*SSB at the replication fork in a sequential manner. However, considering our evidence that *Ec*SSB can rapidly dissociate along the entire substrate, it is also plausible that active displacement of *Ec*SSB by DNA pol combined with the ability of *Ec*SSB to slide along the ssDNA (32) increases the *Ec*SSB density on the remaining template strand. This, in turn, produces a transiently oversaturated *Ec*SSB–ssDNA complex, triggering stimulated unwrapping events followed by stimulated dissociation events distributed along the template strand, which could allow faster displacement of *Ec*SSB allowing polymerization to proceed at a faster rate. Once the DNA template available for binding has decreased such that there is more than one tetramer per 65 or 35 nt, this necessarily forces some protein to unwrap. This unwrapped state, which we were able to isolate using high force and protein concentration or mutation of *Ec*SSB to prevent tetramerization, is extremely unstable under physiological conditions. Thus, neighboring proteins stimulate the unwrapping and subsequent dissociation of their neighbors, until enough substrate is released for the stable wrapping of all remaining *Ec*SSB. Thus, this unwrapped state of *Ec*SBB is not observed under equilibrium conditions, even though it necessarily must exist as a transition state through which *Ec*SSB first binds disordered free ssDNA before wrapping. Importantly, the protein overcrowding on the template strand can be resolved by dissociation of any given *Ec*SSB along the ssDNA fragment, allowing for faster *Ec*SSB dissociation to accommodate the rapid pace of DNA replication, as discussed above. It is also consistent with measured *Ec*SSB dissociation following an exponential function when destabilized by force (Figure 4), structural inhibition of wrapping (Figure 5), or displacement by RecA filaments (Figure 7), instead of a linear function that would result from sequential dissociation. Additionally, previous experiments with fluorescently labeled *Ec*SSB similarly observed tetramers dissociating at increasing rates proportional to free protein concentrations (35). As the ssDNA template gets smaller, this dynamic process may continue and self-regulate the *Ec*SSB density efficiently to allow the DNA pol to proceed while ensuring maximal template coverage at any given time. The proposed mechanism is entirely based on *Ec*SSB’s competitive binding mechanism to ssDNA, and its nearest neighbor interactions that allow oversaturation while stimulating dissociation. We expect future studies utilizing a wide array of *Ec*SSB mutants will provide further insights into the nature of competitive binding characterized in this work, and its relationship to previously observed binding cooperativity mediated by the unstructured C-terminal tails.

## DATA AVAILABILITY

The data that support the findings of this study are available from the corresponding author upon reasonable request.

## Supplementary Material

gkaa1267_Supplemental_File
