# Supplementary material for: Multiprotein E. coli SSB–ssDNA complex shows both stable binding and rapid dissociation due to interprotein interactions
Source: Nucleic Acids Res. 2021 Jan 12;49(3):1532–49. doi: 10.1093/nar/gkaa1267 (PMC7897507; doi:10.1093/nar/gkaa1267)
Supplement: gkaa1267_Supplemental_File [file gkaa1267_supplemental_file.pdf]

Supplementary Information

Multiprotein *E. coli* SSB-ssDNA complex shows both stable binding and rapid dissociation due to interprotein interactions

M. Nabuan Naufer[1], Michael Morse[1], Guðfríður Björg Möller[1], James McIsaac[2], Ioulia Rouzina[3], Penny J. Beuning[2], Mark C. Williams[1*]

[1]Department of Physics, Northeastern University, Boston, MA, 02115
[2]Department of Chemistry and Chemical Biology, Northeastern University, Boston, MA, 02115
[3]Department of Chemistry and Biochemistry, Ohio State University, Columbus, OH 43210
[*]Corresponding author, ma.williams@northeastern.edu

Supplementary Method 1	Explicit analytical description of the kinetics and equilibrium of *Ec*SSB binding and competition between its different states on ssDNA
Supplementary Method 2	Numerical solutions of the two-step competitive binding model
Supplementary Figure S1	Observations of single wrapping/unwrapping events at 12 pN
Supplementary Figure S2	Observations of single wrapping/unwrapping events at 7 pN
Supplementary Figure S3	Exponential fits to individual phases of experiments
Supplementary Figure S4	Force-jump experiments with *Ec*SSB and ssDNA
Supplementary Figure S5	AFM imaging of *Ec*SSB binding short ssDNA segments
Supplementary Figure S6	*Ec*SSB H55Y mutant at high concentrations exhibits partial tetramerization
Supplementary Figure S7	Impact of Magnesium on *Ec*SSB binding dynamics

Supplementary Method 1. Explicit analytical description of the kinetics and equilibrium of *Ec*SSB binding and competition between its different states on ssDNA.

Kinetics of EcSSB-ssDNA complexes during the protein concentration jump cycles.

The bind-wrap transition followed by the unwrap-bind transition is a complicated process with no explicit analytical solution. However, our understanding of the underlying elementary processes allows us to express the main component of each observed rate through the elementary reaction rates of this system. As discussed in the main text, at 12 pN these transitions can be primarily denoted with the following kinetic scheme.

$$EcSSB_{unbound} \underset{k_{-b}}{\overset{ck_b}{\rightleftharpoons}} EcSSB_8 \underset{k_{-w}}{\overset{k_w}{\rightleftharpoons}} EcSSB_{17}. \qquad \text{S1}$$

Here, ck_b and k_{-b} are the forward and reverse rates of free protein binding and k_w and k_{-w} are the forward and reverse rates of bound protein wrapping the ssDNA substrate. Experimentally, we investigate this system by varying the free protein concentration, *c*, that presumably mimics the variations in *Ec*SSB concentration in the bacterial cell due to the fluctuations in protein expression and the availability of transient ssDNA during genomic maintenance. For the sake of simplicity, we derive the analytical expressions describing the kinetics and equilibrium of the three-state system as observed at a template tension of 12 pN where primarily $Ec\text{SSB}_8$ and $Ec\text{SSB}_{17}$ states coexist. At lower forces the coexistence of several higher order wrapped *Ec*SSB states would make deconvolving the system into its fundamental reaction steps mathematically challenging. However, the kinetic scheme described here is qualitatively consistent at lower template tensions where more than two *Ec*SSB states coexist. For example, at a template tension of 7 pN we observe the same qualitative behavior (Fig. 4), but the model would have to be expanded to incorporate higher order states such as $Ec\text{SSB}_{35}$, and $Ec\text{SSB}_{56}$ in addition to the $Ec\text{SSB}_8$ and $Ec\text{SSB}_{17}$ states (Fig. S2). As described in the text we find that the *Ec*SSB dissociation and unwrapping kinetics depends on the degree of ssDNA saturation. When there are more *Ec*SSB on the ssDNA substrate than can be accommodated in a wrapped state, this is defined to be *Ec*SSB oversaturated. Thus, the net dissociation and unwrapping rates are given by,

$$k_{-b} = k^0_{-b} + k^s_{-b} \quad and \quad k_{-w} = k^0_{-w} + k^s_{-w}. \qquad \text{S2}$$

Here, the superscripts 0 and s represent an *Ec*SSB -unsaturated and -oversaturated complex, respectively.

The explicit expressions for the observed bind-wrap ($k_{b,w}$), bind-unwrap ($k_{b,-w}$), and unbind-wrap ($k_{-b,w}$) rates in terms of the four fundamental forward and reverse kinetic rates are derived as a function of the free *Ec*SSB concentration in solution. During the bind-wrap transition *Ec*SSB binds and subsequently wraps an unsaturated ssDNA in series, and therefore the observed rate, $k_{b,w}$ can be estimated by,

$$\frac{1}{k_{bw}} = \frac{1}{ck_b} + \frac{1}{k_w} \;\; \rightarrow \;\; k_{bw}(c) = \frac{ck_b k_w}{ck_b + k_w} = \frac{k_w}{1 + \frac{k_w}{ck_b}}. \qquad \text{S3}$$

Fitting the observed bind-wrap transition (Fig. 3C blue) to Eq. S3 yields, k_b=0.15 $nM^{-1} \cdot s^{-1}$ and k_w=1.4 s^{-1}

The subsequent bind-unwrap transition occurs as the ssDNA substrate becomes *Ec*SSB oversaturated. Thus, the observed rate $k_{b,-w}$ for this transition is given by,

$$\frac{1}{k_{b,-w}} = \frac{1}{ck_b} + \frac{1}{k_{-w}} \;\; \rightarrow \;\; \frac{ck_b k_{-w}}{ck_b + k_{-w}} = \frac{k_{-w}}{1 + \frac{k_{-w}}{ck_b}} \approx \frac{k^s_{-w}}{1 + \frac{k^s_{-w}}{ck_b}} \qquad \text{S4}$$

Experimentally, we do not observe unwrapping on an unsaturated ssDNA substrate at 12 pN as evidenced by the maximally wrapped *Ec*SSB-ssDNA complex being stable >100 s upon the removal of free *Ec*SSB in the solution (Fig. 1D&E and Fig. 3A, magenta. We observe the unwrapping transition due to nearest neighbor interactions at sufficiently high protein concentrations when the ssDNA substrate is oversaturated (Fig. 1C). For this reason, the final approximation in Eq. S4 holds because the unwrapping rate on a saturated ssDNA substrate (stimulated unwrapping) must be at least an order of magnitude faster than that of on an unsaturated ssDNA substrate. Accordingly, at saturation stimulated unwrapping becomes the rate-limiting step and the fit yields, k^s_{-w}=0.095 s^{-1} (Fig. 3C, orange).

Finally, the rate of the unbind-wrap transition in which an *Ec*SSB_8 dissociation is followed by the further wrapping of a neighboring protein is given by,

$$\frac{1}{k_{-bw}} = \frac{1}{k^s_{-b}} + \frac{1}{k_w} \;\; \rightarrow \;\; k_{-bw}(c) = \frac{k^s_{-b}}{1 + \frac{k^s_{-b}}{k_w}} \approx k^s_{-b}. \qquad \text{S5}$$

As the unbind-wrap transition occurs on an *Ec*SSB-oversaturated ssDNA substrate, k^s_{-b} represents the stimulated dissociation rate due to nearest neighbor interactions. Therefore, here we measure the rate of *Ec*SSB dissociation from an oversaturated ssDNA substrate to be k^s_{-b}=0.11 s^{-1} (Fig. 3C, magenta). Note that we directly measure a high force (wrapping prohibited) dissociation rate of *Ec*SSB from an unsaturated ssDNA to be k^0_{-b}=0.017 s^{-1} (Figs. 4C and S4). Therefore, the stimulated unwrapping and stimulated dissociation on a saturated ssDNA substrate are at least an order of magnitude faster than the equivalent processes on an unsaturated substrate.

Competition of different EcSSB states within the equilibrated oversaturated EcSSB-ssDNA complex.

From the association and dissociation rates observed on an unsaturated ssDNA substrate we can determine the equilibrium dissociation constant, K_{db} of *Ec*SSB binding to an $Ec\text{SSB}_8$ state to be,

$$K_{db} = \frac{k_{-b}^0}{k_b} = \frac{0.014\, s^{-1}}{0.18\, nM^{-1} s^{-1}} = 0.08\, nM, \qquad \text{S6}$$

and the corresponding free energy to be,

$$G_{b,0}(k_B T) = \ln\left(\frac{c}{K_{db}}\right). \qquad \text{S7}$$

In the concentration-jump experiments (Fig. 3) the *Ec*SSB-ssDNA complex remains oversaturated between the bind-wrap transition through the quasi-equilibrium bind-unwrap transition up until the maximum wrapping after dissociation during the unbind-wrap transition. ($\Delta X_{b,w} \rightarrow \Delta X_{b,-w} \rightarrow \Delta X_{-b,w}$ in Fig. 3A-B). The equilibrium complex extension upon the bind-unwrap transition, $\Delta X_{b,-w}$ is defined by the fractions of saturated ssDNA engaged with either of the $Ec\text{SSB}_8$ (Θ_b), or $Ec\text{SSB}_{17}$ (Θ_w) states where,

$$\Delta X = -[\Delta x_b \Theta_b + \Delta x_w \Theta_w]. \qquad \text{S8}$$

Here Δx represents the corresponding ssDNA length-change associated with each binding state. We can express these equilibrium competing fractions of *Ec*SSB states analytically in terms of their binding free energies and fundamental reaction rates as a function of free *Ec*SSB concentration (c) at 12 pN. We assume that in equilibrium at 12 pN the ssDNA substrate is bound by either $Ec\text{SSB}_8$ (B), or $Ec\text{SSB}_{17}$ (W) *Ec*SSB states. Even though the free energy of W state per protein, $G_b + G_w$ is larger than the free energy of B state per protein G_b, a single *Ec*SSB in W can be substituted by multiple B complexes with the smaller binding free energy due to different binding site sizes (N), where $N_w/N_b = 1/\eta > 1$. The equilibrium B and W fractions of associated with ssDNA substrate per N_w nt is given by,

$$\eta.\bar{B} + \overline{W} = 1 \qquad \text{S9}$$

Θ_w and Θ_b are the ssDNA fractions engaged with the W and B states, respectively. Therefore,

$$\Theta_w = \overline{W} = \frac{e^{(G_b+G_w)/k_B T}}{Z} = \frac{1}{1+e^{-\delta G/k_B T}}, \qquad \text{S10}$$

and

$$\Theta_b = \eta.\bar{B} = \frac{e^{(G_b/\eta)/k_B T}}{Z} = \frac{1}{1+e^{\delta G/k_B T}}, \qquad \text{S11}$$

where

$$Z = e^{(G_b+G_w)/k_BT} + e^{(G_b/\eta)/k_BT} \quad and \quad \delta G = G_w - G_b(1/\eta - 1). \qquad \text{S12}$$

δG is the free energy difference per N_w of ssDNA length between W- and B- saturated states. The midpoint of the transition with equal fractions of ηB=W=1/2 occurs when

$$\delta G = 0 \quad or \quad G_w = G_b(1/\eta - 1) \qquad \text{S13}$$

The later condition defines the midpoint free *Ec*SSB concentration, $c^*(F)$ at which *Ec*SSB$_8$ and *Ec*SSB$_{17}$ states are equally probable where,

$$c^* = \frac{k_{-b}}{k_b} . \left(\frac{k_w}{k^s_{-w}}\right)^{\eta/1-1} = K_{db}\left(\frac{k_w}{k^s_{-w}}\right)^{\eta/1-1} \qquad \text{S14}$$

The equilibrium fractions of ssDNA associated with the W and B state as given by Eq. S10 and Eq. S11 can be now written as,

$$\Theta_w = \overline{W} = \frac{1}{1 + \frac{k^s_{-w}}{k_w} . \left(\frac{K_{db}}{c}\right)^{1/\eta-1}} = \frac{1}{1 + \left(\frac{c}{c^*}\right)^{1/\eta-1}} \qquad \text{S15}$$

$$\Theta_b = \eta\overline{B} = \frac{1}{1 + \frac{k^s_{w}}{k_{-w}} . \left(\frac{K_{db}}{c}\right)^{1/\eta-1}} = \frac{1}{1 + \left(\frac{c^*}{c}\right)^{1/\eta-1}} \qquad \text{S16}$$

We model the observed $\Delta X_{b,-w}$ ($\equiv \Theta_b$) substituting the experimentally determined, k_w (=1.3 s^{-1}), and k^s_{-w} (=0.1 s^{-1}) by letting η and K_{db} be free parameters (Fig. 2C, solid line). The fit yields ~ 0.6, which is consistent with N_w and N_b being ~17 nt and ~8 nt, respectively. The fit yields K_{db}~ 0.04 nM, which is comparable to the measured K_{db} (= 0.08 nM, Eq. S6). This suggests that the on/off kinetics of *Ec*SSB binding to unsaturated ssDNA is consistent with the competitive titration data in Fig. 2C. The transition midpoint concentration, c^* at which the bound *Ec*SSB$_8$ displaces the wound *Ec*SSB$_{17}$ can be estimated to be, ~4 nM. This value of c* is much higher than the actual K_{db} (~ 0.08 nM) of *Ec*SSB$_8$ due to the competition with wrapped *Ec*SSB states. The other manifestations of the same competition are the much faster stimulated dissociation and stimulated unwrapping events on an oversaturated ssDNA, as discussed in the main text.

Supplementary Method 2. Numerical solutions of the two-step competitive binding model.

To test the proposed three-state kinetic model, we first derive differential equations based on the kinetic scheme described in Eq. 1, which determines the rate of change in the fraction of ssDNA substrate that is in the unbound (Θ_0), bound (Θ_b), and wrapped (Θ_w) states as a function of the fundamental rates of *Ec*SSB binding, dissociation, wrapping, and unwrapping.

$$\begin{aligned}
\frac{d\Theta_0}{dt} &= \Theta_b k_{-b} - \Theta_0 c k_b + \Theta_w k_{-w}(1-\eta) - \Theta_0\Theta_b k_w(\eta^{-1}-1) \\
\frac{d\Theta_b}{dt} &= \Theta_0 c k_b - \Theta_b k_{-b} + \Theta_w \eta k_{-w} - \Theta_0\Theta_b k_w \\
\frac{d\Theta_w}{dt} &= \Theta_0\Theta_b \eta^{-1} k_w - \Theta_w k_{-w}
\end{aligned} \tag{S17}$$

The terms associated with transitions between the unwrapped and wrapped states has a dependency on the ratio of the binding site sizes (η) as the wrapping of a protein increases the amount of the ssDNA it occupies. We assume η = 8 nt / 17 nt here. Furthermore, the exact value k_b depends on the defined binding site size, as a longer ssDNA segment will become bound by a protein more quickly than a shorter segment. We use a binding site size of 17 nt (the assumed site size at equilibrium) to define k_b. Note that we are specifically modelling *Ec*SSB binding dynamics at 12 pN, where *Ec*SSB_{17} is the only stable wrapped state. Modeling higher order wrapped states would require adding additional terms for the occupancy of these states. We substitute fundamental rates as determined by our concentration dependent binding experiments (Fig. 3 and Table 1) and solve the differential equations using the Euler method for small discrete time steps (1 ms). This yields the fractions of ssDNA in each state over time for a given free concentration of protein. Additionally, the free protein concentration is abruptly changed to zero after the incubation step is complete to simulate the concentration-jump experiments. Finally, the occupancy of each state over time is converted into an observable change in ssDNA extension for direct comparison with experimental data. The total extension change as a function of time due to *Ec*SSB binding, ΔX (t), is given by

$$\Delta X(t) = -\left[\Delta X_b \Theta_b(t) + \Delta X_w \Theta_w(t)\right] \tag{S18}$$

where ΔX_b and ΔX_w are the extension changes associated with the bound $EcSSB_8$ and wrapped *Ec*SSB_{17} states, respectively. Accordingly, we use N_w to be 17 nt and the associated change in extension to be 0.08 nm/nt, as observed in Fig. 3B. The minimum extension change we observe at 50 nM (0.02 nm/nt) serves as an upper bound to the extension change associated with the bound *Ec*SSB_8 state. Therefore, we chose N_b to be 8 nt (supported by AFM experiment, Fig. S5) and the associated extension change to be 0.015 nm/nt (supported by the monomeric *Ec*SSB mutant H55Y, Fig. S6). Using these experimental derived rates and amplitudes, the model correctly reproduces the time dependent binding curve (Fig. 2B) and equilibrium between the

wrapped and unwrapped *Ec*SSB states (Fig. 2C) over the range of free protein concentrations observed. As the unwrapping rate of an *Ec*SSB on an unsaturated ssDNA (k^0_{-w}), is too slow to be measured in our system, we use k^0_{-w}= 0.01 s^{-1} in the simulation that do not take stimulated unwrapping into account (Fig. 6C, dashed blue line), This estimation is based on the experimental observation that the maximally wrapped *Ec*SSB is stable over >100 s in the absence of free *Ec*SSBs in the solution at 12 pN (Fig. 1D&E and Fig. 3A, magenta).

Supplementary Figures

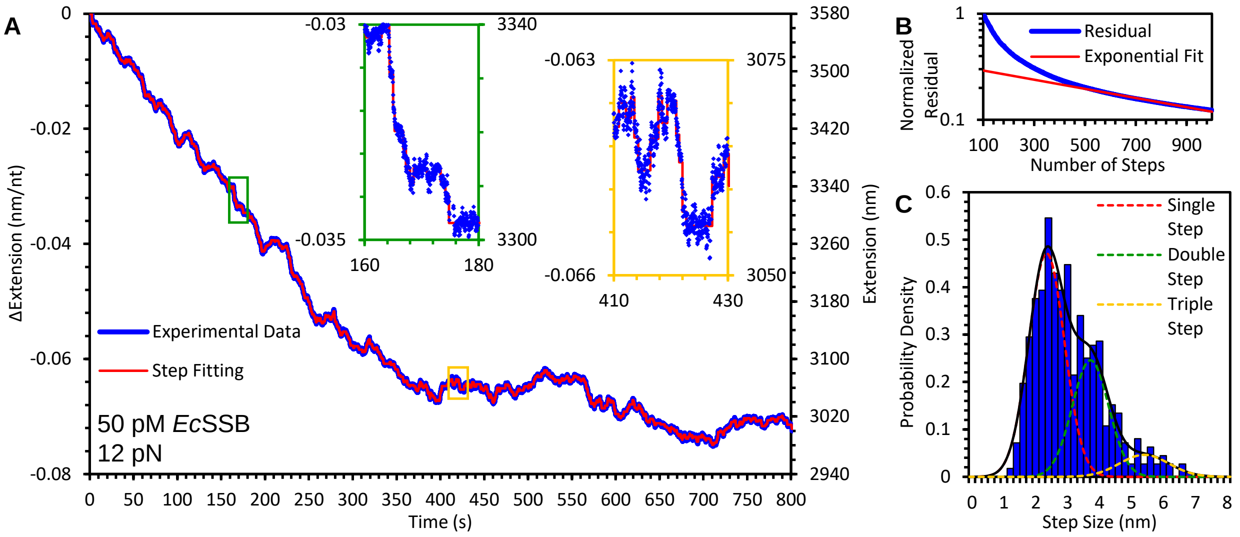

Figure S1: Observations of single wrapping/unwrapping events at 12 pN. (A) Extension-time profile of an ssDNA molecule incubated with 50 pM *Ec*SSB (blue) is fitted to a step resolving function in MATLAB (red). The total number of steps is predefined (100-1000 steps tested, 600 steps shown here), while the amplitude and timing of each step is determined by least square minimization. Initially decreasing steps are observed indicating *Ec*SSB wrapping events (green inset). As the system reaches equilibrium, steps in both directions are observed, indicating dynamic equilibrium of wrapping and unwrapping events (yellow inset). (B) The total residual of the step function fit strictly decreases as the number of steps are increased (blue). Initially, the improvement of the fit is rapid as added steps are localized to real extension drops present in the data. Past a certain threshold (~600 steps), the residual will continue to decay at a much slower exponential rate (red) as newly added steps merely fit to the random noise present in the data. This threshold value is used for the final fit to determine the ssDNA compaction associated with individual *Ec*SSB wrapping events. (C) A histogram of the absolute size of wrapping/unwrapping events exhibits an asymmetric distribution with a peak between 2 and 3 nm and a long tail displaying larger ssDNA extension changes. Since the 8.1 knt ssDNA substrate can accommodate 100s of proteins and the limited temporal resolution of the measurements, some detected steps in extension are due to multiple wrapping events occurring nearly concurrently. Fitting a triple gaussian to the step distribution without constraints on the fitting parameters returns three peaks evenly spaced (~2.4, ~3.7, and ~5.4 nm) with larger events displaying reduced amplitude. These results are consistent with each wrapping event reducing ssDNA extension by ~2 nm and a smaller chance of multiple events occurring concurrently. Note, while lower concentrations of *Ec*SSB would allow for better temporal resolution due to less frequent wrapping events, 50 pM was the lowest concentration of *Ec*SSB where we reliably observed near saturation of the ssDNA substrate during the timescale of the experiments. However, lower concentration experiments may be unreliable, as the very dilute protein solution may not be entirely stable at room temperature in low salt buffer over the ~1-hour timescale that would be required to observe saturated binding.

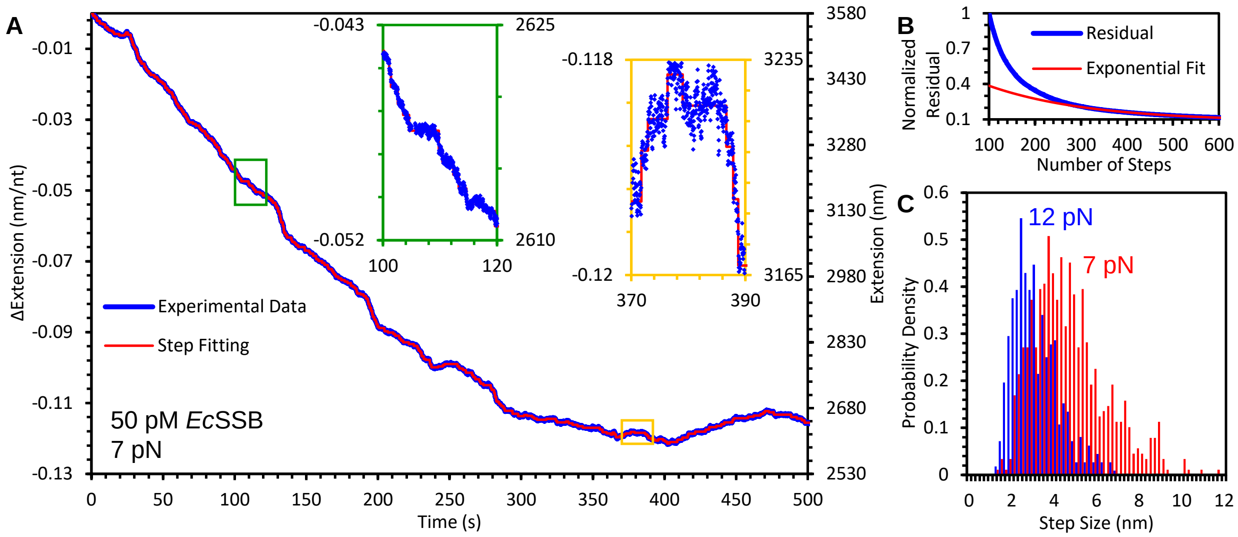

Figure S2: Observations of single wrapping/unwrapping events at 7 pN. (A) The analyses described in Fig. S1 here is repeated on an Extension-time profile obtained at ssDNA held at 7 pN with 50 pM *Ec*SSB. Data are shown in blue and the step finding fit in red. Insets show steps that represent exclusively wrapping events, and wrapping events followed by unwrapping events, respectively. (B) The residuals from the step fitting procedure shows that that these data can be well fit with significantly fewer steps than the 12 pN data, suggesting fewer proteins are required to bind and wrap the ssDNA at saturation, consistent with the protein assuming a more wrapped conformation with a larger associated binding site size. (C) A comparison of the absolute step sizes of EcSSB binding ssDNA under 12 and 7 pN tension shows similar asymmetric distributions, with a larger average step size at reduced tension.

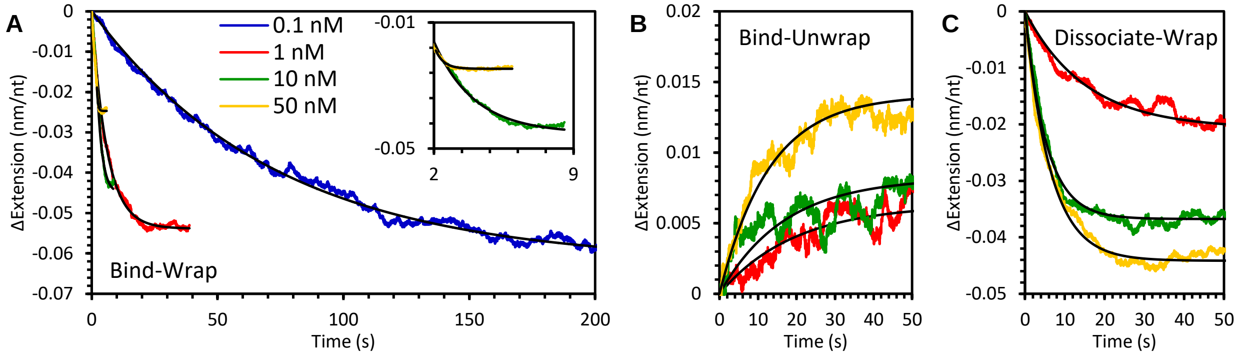

Fig S3. Exponential fits to individual phases of experiments. Extension changes for experiments with each concentration associated with the bind-wrap (A), bind-unwrap (B), and dissociate-wrap (C), are all well fit by single exponential equations. Note, the fits in (A) start two seconds after protein enters the channel to ensure maximum binding and ends at the minimum in extension before the subsequent biphasic increase in extension. The inset magnifies the high concentration data, in which ssDNA reaches a minimum extension within seconds of the introduction of free protein.

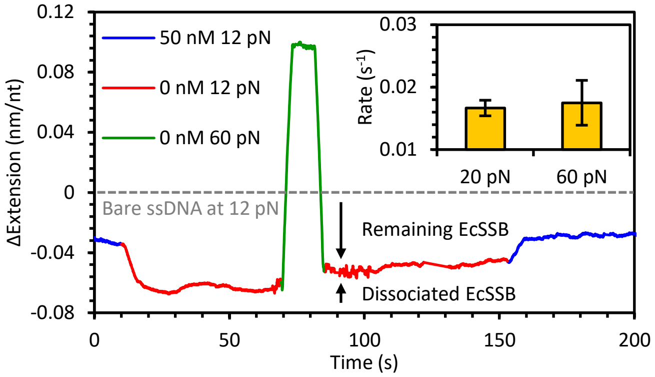

Figure S4: Force-jump experiments with *Ec*SSB and ssDNA: After the *Ec*SSB-ssDNA complex is saturated upon initial incubation (blue, bind-wrap transition is not shown), and the free protein is removed (unbind-wrap shown in red) at 12 pN, the *Ec*SSB-ssDNA complex equilibrates at the maximally wrapped state with no dissociation observed. Then the applied force on the template is rapidly increased, held at 60 pN and returned to 12 pN during a ~15 s timescale. Most protein remains bound during this transition and is able to rewrap the ssDNA at 12 pN, as evidenced by the mostly preserved change in extension (with respect to protein-free ssDNA). Additionally, reintroducing free protein (at t~150 s), results in an increase in extension that is consistent with the rebind-unwrap process (Fig. 3), rather than a biphasic binding curve observed with *Ec*SSB binding to protein-free ssDNA, reinforcing that most *Ec*SSB remains bound. The change in ssDNA extension before and after the 60 pN force spike is used to estimate the amount of protein that dissociated at high force and calculate the rate of *Ec*SSB dissociation in the absence of wrapping events (k_{-b}). This value (inset) is equal to the rate of dissociation we directly observe at 20 pN.

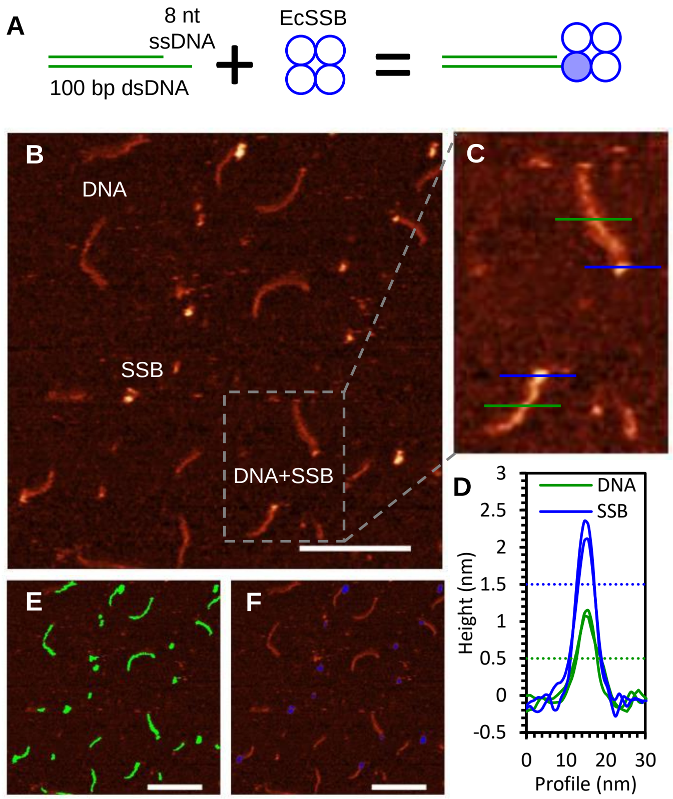

Fig S5. AFM imaging of *Ec*SSB binding short ssDNA segments. (A) A DNA construct consisting of 100 bp of dsDNA with an 8 nt poly(dT) ssDNA overhang is incubated at equimolar concentrations (5 nM each). The schematic shows that the ssDNA overhang can only accommodate a single OB domain of *Ec*SSB. (B) These *Ec*SSB-DNA complexes are deposited on a treated mica surface and imaged using AFM (scale bar 100 nm). The DNA constructs appear as faint lines (ssDNA region cannot be observed distinctly from the dsDNA) and *Ec*SSB proteins appear as bright globules. *Ec*SSB tetramers localized at one end of the DNA construct indicate ssDNA binding. (C) Inset shows representative images of *Ec*SSB-DNA complexes. (D) Height profiles of dsDNA (green lines in (C)), and *Ec*SSB (blue lines in (C)), show that the measured maximum height of *Ec*SSB is approximately twice that of dsDNA. Therefore, a threshold of 0.5 nm (dotted green line), and 1.5 nm (dotted blue line) distinguish dsDNA, and *Ec*SSB, respectively, from the background. (E-F) Applying these thresholds to the entire image captures either both dsDNA and *Ec*SSB (green, threshold=0.5 nm) or *Ec*SSB only (blue, threshold=1.5 nm).

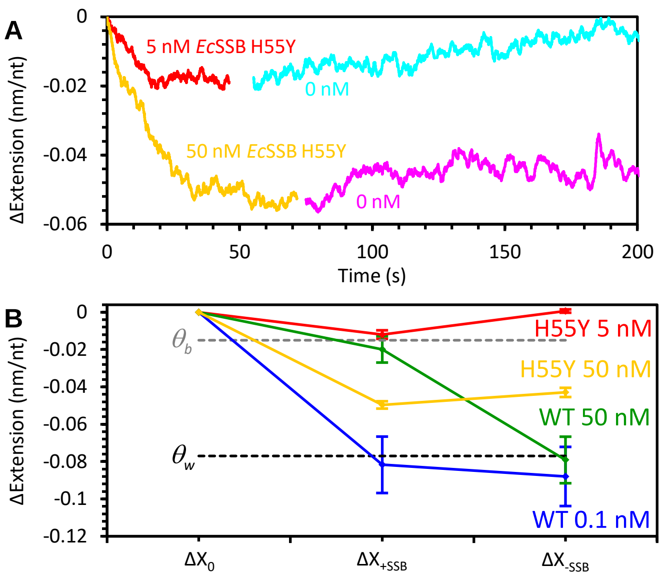

Fig S6. EcSSB H55Y mutant at high concentrations exhibits partial tetramerization. (A) When ssDNA held at 12 pN is incubated with 50 nM H55Y mutant *Ec*SSB, the ssDNA compaction is greatly increased (yellow) and only partial dissociation is observed when the free protein is removed (magenta), as compared to 5 nM incubation (red, data replotted from Fig. 5) and dissociation (cyan), where compaction is minimized and dissociation is complete. (B) Plotting these equilibrium extension values after incubation and dissociation shows that high concentration H55Y mutant *Ec*SSB exhibits behavior more similar to WT *Ec*SSB, suggesting partial tetramerization of free protein, as compared to low concentration H55Y mutant *Ec*SSB, which behaves like a pure monomer.

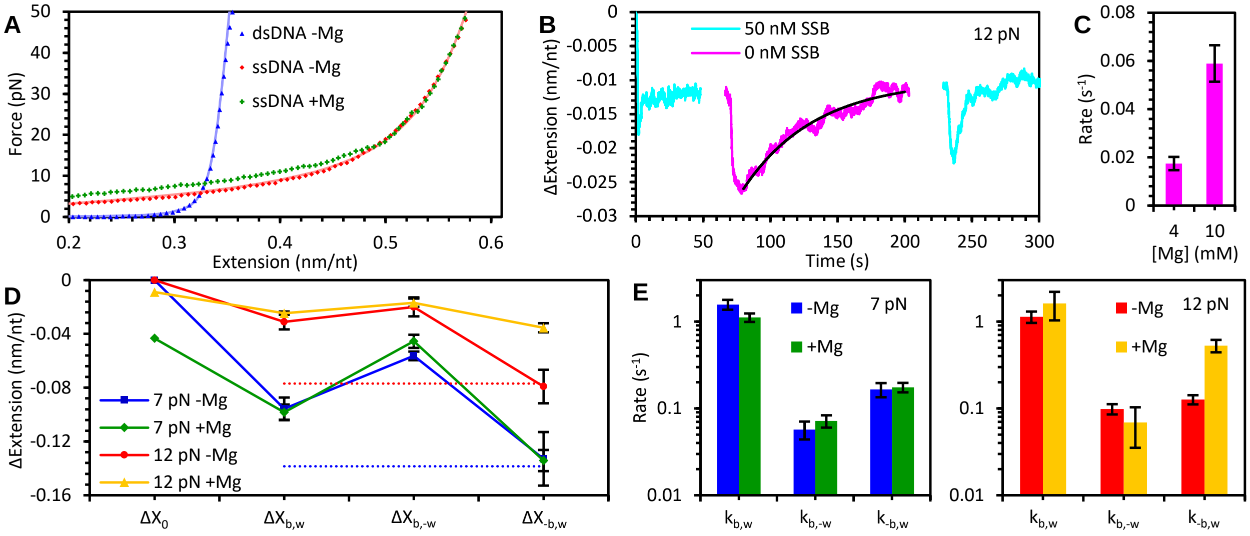

Figure S7: Impact of Magnesium on *Ec*SSB binding dynamics. (A) Magnesium (4 mM Mg^{2+}) stabilizes the local secondary structures of ssDNA (such as hairpins), resulting in apparent reduction in extended length (green) at forces <20 pN. At forces > 20 pN the secondary structures are eliminated rendering similar ssDNA extension profiles in the presence (red) and absence of Mg^{2+}. (B) In the presence of Mg^{2+}, *Ec*SSB still exhibits a biphasic binding pattern (cyan). After removing the free *Ec*SSB from the solution, the unbind-wrap process is followed by direct dissociation at 12 pN of most bound protein (magenta). This is similar to what is observed at 20 pN in the absence of Mg^{2+} (Fig. 4). (C) The rate of the direct dissociation measured here increases as the Mg^{2+} concentration is increased. (D) The total change in extension due to *Ec*SSB binding (50 nM) is similar both in the presence and absence of magnesium. Note that the length decrease of the protein-free ssDNA due secondary structures in the presence of Mg^{2+} is taken into account (ΔX_0 is non-zero). (E) The rates associated with each phase of the *Ec*SSB binding (as described in Fig. 3) are similar in the presence and absence of Mg^{2+}. Overall the results suggest that the presence of Mg^{2+} destabilizes the low order *Ec*SSB wrapped conformations, and enhances *Ec*SSB dissociation from the unsaturated complex when wrapping is unfavorable due to template tension.
